# Socioeconomic status and lifestyle as factors of multimorbidity among older adults in China: results from the China Health and Retirement Longitudinal Survey

**DOI:** 10.3389/fpubh.2025.1586091

**Published:** 2025-07-30

**Authors:** Wei Gong, Xiaoxiao Hu, Huimin Cui, Yuxin Zhao, Hong Lin, Peng Sun, Jianjun Yang

**Affiliations:** ^1^Public Health School, Ningxia Medical University, Yinchuan, China; ^2^Key Laboratory of Environmental Factors and Chronic Disease Control, Yinchuan, China; ^3^School of Medical Information and Engineering, Ningxia Medical University, Yinchuan, China; ^4^School of Nursing, Ningxia Medical University, Yinchuan, China; ^5^Institute of Medical Science and Technology, Ningxia Medical University, Yinchuan, China

**Keywords:** machine learning, multimorbidity, Chinese older adult, XGBoost, SHAP, predictive model

## Abstract

**Background:**

Multimorbidity is increasingly prevalent among older adults and poses significant challenges to public health systems. While previous studies have highlighted the role of individual behaviors, the complex interaction between lifestyle factors and socioeconomic status (SES) in multimorbidity remains unclear.

**Methods:**

Using nationally representative data from the China Health and Retirement Longitudinal Study (CHARLS), we developed predictive models to identify key determinants of multimorbidity among individuals aged ≥60 years. A total of 34,755 participants were included, and 17 features related to demographics, SES, and lifestyle were selected via LASSO regression. Eight machine learning algorithms including logistic regression, decision tree, naive Bayes, neural network, support vector machine, random forest, XGBoost and Bayesian Ridge Regression were applied to build predictive models. Model performance was evaluated using AUC, accuracy, precision, recall, F1-score, RMSE, and decision curve analysis (DCA). SHapley Additive exPlanations (SHAP) were used to interpret model outputs.

**Results:**

XGBoost achieved the best predictive performance (AUC = 0.788 on the test set), outperforming both linear and non-linear models across most evaluation metrics. SHAP analysis revealed that education level, activities of daily living (ADL), work status, self-assessed health status, and per capita income were the top factors associated with of multimorbidity. Subgroup analyses showed variated associations by age and sex, with psychological and geographic factors playing a larger role among those aged ≥80.

**Conclusion:**

This study demonstrated the feasibility and interpretability of using machine learning to model complex risk patterns of multimorbidity. Socioeconomic and functional variables were dominant factors associated with multimorbidity, suggesting structural roots of health inequality. These findings offered empirical and theoretical support for early risk stratification and targeted public health interventions aimed at mitigating multimorbidity in aging populations.

## Graphical abstract

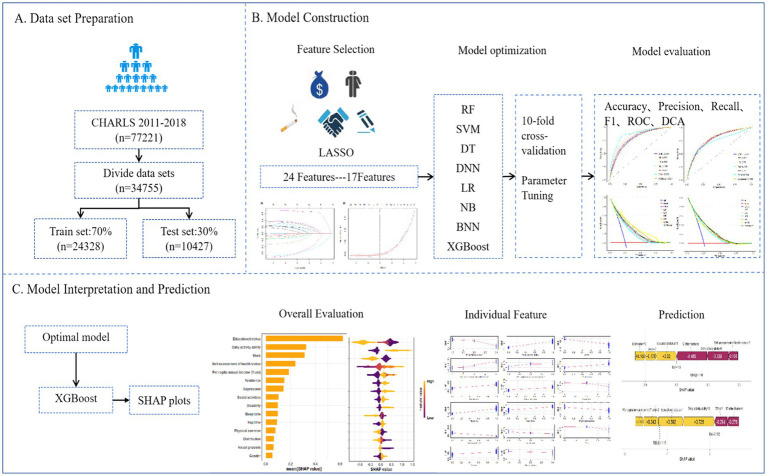

## 1 Introduction

With the acceleration of global population aging and profound shifts in lifestyle, multimorbidity, the coexistence of two or more chronic conditions has become increasingly prevalent, especially among older adults. It now poses a major challenge for global public health systems (1, 2). Multimorbidity prevalence rates as high as 55–98% among those aged 60 and above (3). It accelerates physical decline and increases the risk of mental health issues and mortality (4, 5). Therefore, identifying high-risk populations and designing effective intervention strategies are critical for improving health equity in aging populations.

Multimorbidity is shaped not only by biomedical factors but also by broader social determinants, including lifestyle behaviors, environmental exposures, and social structures (5, 6). Currently, China is in the process of urbanization, where socioeconomic status differences lead to health disparities among the older adult, with a weaker socioeconomic status negatively impacting older adult health (7, 8). Lifestyle, public services, and social psychological factors can mitigate the direct impact of socioeconomic status on older adult health to some extent. Previous studies on lifestyle and chronic diseases have often focused on individual-level factors (9–11), overlooking the broader social context in which healthy behaviors occur. Key socioeconomic status (SES) indicators, such as education level, occupational status, and income are crucial (12, 13). However, how these factors interact within China’s unique social transformation remains underexplored.

While previous studies have identified demographic and clinical predictors of multimorbidity (14, 15), few have integrated sociological theory to examine how macro-level social change interacts with individual health behaviors. ML offers new analytical pathways by capturing non-linear, multidimensional relationships between social structure and health outcomes, enabling deeper insights into the social roots of chronic disease. Traditional statistical methods used in prior studies often fall short in capturing non-linear relationships and complex interactions among variables. In contrast, machine learning (ML) has emerged as a powerful tool for addressing complex problems and has been increasingly applied in healthcare research (16–19). To enhance interpretability and policy relevance, Shapley Additive Explanations (SHAP) are increasingly used to visualize and explain contributions of variables in machine learning models (20–23).

Drawing on data from the China Health and Retirement Longitudinal Study (CHARLS), this study integrated variables related to socioeconomic status, lifestyle, and self-reported health, and applied a suite of machine learning algorithms to construct interpretable predictive models. Our aim was to systematically identify key social and behavioral determinants of multimorbidity in older Chinese adults. This research contributed to the growing literature by providing theoretical and empirical insights for early identification of high-risk groups and offered actionable evidence for the development of more targeted and equitable health policies in the context of population aging in China.

## 2 Materials and methods

### 2.1 Data source

This study is based on national data from the China Health and Retirement Longitudinal Survey (CHARLS) conducted by the National Development Research Institute of Peking University^1^. This study is an ongoing community-based cohort study of a nationally representative sample of Chinese residents aged 45 and older. To ensure best practice and internationally comparable results, CHARLS is coordinated with leading international research in the Health and Retirement Research (HRS) model. A stratified multi-stage random sampling strategy was adopted. Follow-up visits have been conducted every 2 years since 2011, with the most recent follow-up in 2020, and comprehensive and detailed information on demographics, socioeconomic status, biomedical measurements, health status, and functioning were collected. To ensure sample representativeness, the CHARLS baseline survey covers 150 countries/regions and 450 villages/urban communities across the country, involving 10,257 households with 17,708 people, and reflects the middle-aged and older adult in China (24).

**Figure 1:**
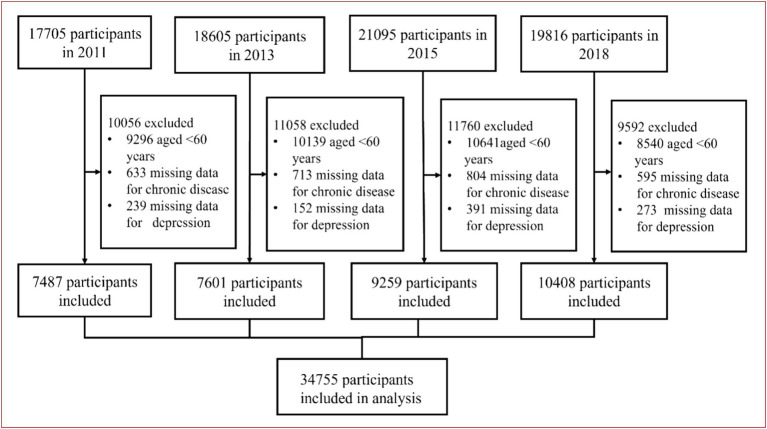
Flowchart of study participant selection.

In this study, we included 77,221 participants from the 2011–2018 study waves, of whom 34,755 were eligible for model development and internal validation. Inclusion criteria: (1) participants aged ≥60 years; exclusion criteria: (1) participants aged <60 years; (2) participants without missing data for chronic disease and depression. The detailed inclusion and exclusion process was shown in Figure 1.

### 2.2 Variable selection and definition

#### 2.2.1 Dependent variable

In the questionnaire, each follow-up visit was asked about new diagnoses by doctors of a set of chronic diseases and the timing of diagnoses of specific conditions, where relevant, current medications and treatments for each specific condition. The survey content of chronic diseases in CHARLS. “have you been diagnosed with [conditions listed below] by a doctor?,” with diseases including hypertension; dyslipidemia (elevation of low-density lipoprotein, triglycerides, and total cholesterol, or a low high-density lipoprotein level); diabetes or high blood sugar; cancer or malignant tumor (excluding minor skin cancers); chronic lung diseases (such as chronic bronchitis or emphysema, excluding tumors or cancers); liver disease (except fatty liver, tumors, and cancer); heart attack (including coronary heart disease, angina, congestive heart failure, or other heart problems); stroke; kidney disease (except for tumor or cancer); stomach or other digestive diseases (except for tumor or cancer); emotional, nervous, or psychiatric problems; memory-related disease (such as dementia, brain atrophy, and Parkinson’s disease); arthritis or rheumatism and asthma (24). We divided multimorbidity into two categories: (1) no multimorbidity (0 ≤ chronic diseases ≤1) and (2) multimorbidity (chronic diseases ≥ 2).

#### 2.2.2 Predictor variables

A preliminary evaluation of predictors related to multimorbidity based on clinical significance and scientific knowledge identified 24 variables as candidate predictors. Specifically, it includes: (1) Demographic characteristics (age, gender, marital status, residence, geographical distribution); (2) Lifestyle (smoking, drinking, disability, vision problem, hearing problem, sleep time, nap time, depression, activities of daily living (ADL), instrumental activities of daily living (IADL), self-assessment of health, physical exercise, physical pain condition, social activities); (3) Socioeconomic variables [educational status, Per capita annual income (Yuan), work, medical insurance, endowment insurance], specific variables are described in Supplementary Table S1.

### 2.3 Statistical methods

#### 2.3.1 Data collection and preprocessing

Variables with missing data exceeding 30% were excluded prior to imputation to avoid introducing excessive bias through imputation (25). For the remaining variables with missing data (<30%), mean imputation using regression models was employed. This method was selected for its computational efficiency and relative stability compared to simpler methods, especially given the large sample size (26, 27). Regression-based mean imputation utilizes the relationships observed in the non-missing data to estimate missing values more accurately than a simple overall mean (28), while remaining less computationally intensive than multiple imputation methods for this high-dimensional dataset (25, 29, 30). The preprocessing steps involved duplicate checking, outlier detection, and standardized variable encoding, all conducted in Python 3.7. The cleaned dataset was randomly split into a training set (70%) and a testing set (30%), ensuring no significant differences in baseline characteristics between the two groups (*p* > 0.05). The training set was used to develop the models, while the test set was reserved for hyperparameter tuning and performance evaluation. To reduce dimensionality and enhance model generalizability, we applied the Least Absolute Shrinkage and Selection Operator (LASSO) method with 10-fold cross-validation to identify the optimal penalty parameter (*λ*), selecting the most predictive features from 22 candidates by minimizing binomial deviance.

#### 2.3.2 Model construction and evaluation

We constructed predictive models using eight machine learning algorithms, each with distinct advantages and limitations. Logistic Regression (LR) is a linear probabilistic model that employs the sigmoid function to estimate binary outcomes; it is computationally efficient and interpretable but limited in modeling non-linear relationships. Random Forest (RF), an ensemble method based on bootstrapped aggregation of decision trees, is robust to overfitting and effective for high-dimensional data, though computationally demanding. Extreme Gradient Boosting (XGBoost) is a scalable and regularized gradient boosting framework that delivers high accuracy and supports parallel computation, but it requires careful hyperparameter tuning. Support Vector Machine (SVM) constructs optimal hyperplanes to maximize class separation, performing well with small samples and non-linear kernels, yet it is sensitive to noise and missing values. Naive Bayes (NB) is a probabilistic classifier grounded in Bayes’ theorem with an assumption of conditional independence among features; it is efficient for high-dimensional sparse data, although performance may decline when this assumption is violated. Decision Tree (DT) models recursively partition data based on criteria such as information gain or Gini index; they are easy to interpret but prone to overfitting. Deep Neural Networks (DNN) can learn complex non-linear relationships through backpropagation and are highly powerful given large datasets, but they require considerable computational resources and are less interpretable. Bayesian Ridge Regression (BRR) applies L2 regularization within a Bayesian framework by introducing a Gamma prior on the coefficients, optimizing hyperparameters via marginal likelihood to automatically control model complexity and avoid overfitting. It produces probabilistic predictions and is suitable for small datasets, yet its applicability is restricted to linear relationships, it is sensitive to prior assumptions, and it has relatively low computational efficiency (31).

All models were trained using 10-fold cross-validation and grid search to optimize hyperparameters, with the primary objective of maximizing the Area Under the ROC Curve (AUC) while controlling model complexity. To evaluate model performance, we applied multiple metrics: Accuracy, Precision, Recall, F1-score, Brier Score, Log Loss. AUC-ROC curves assessed the trade-off between true positive rate (TPR) and false positive rate (FPR). Decision Curve Analysis (DCA) used to estimate the net benefit of each model at various threshold probabilities.

#### 2.3.3 Model interpretability

To enhance interpretability, we employed SHapley Additive exPlanations (SHAP) on the best-performing model (XGBoost). SHAP values quantified the contribution of each feature to individual predictions, while summary plots ranked overall feature importance (32). SHAP dependence plots illustrated non-linear effects and interaction patterns, and force plots were used to explain individual-level predictions, facilitating clinical understanding.

**Figure 2:**
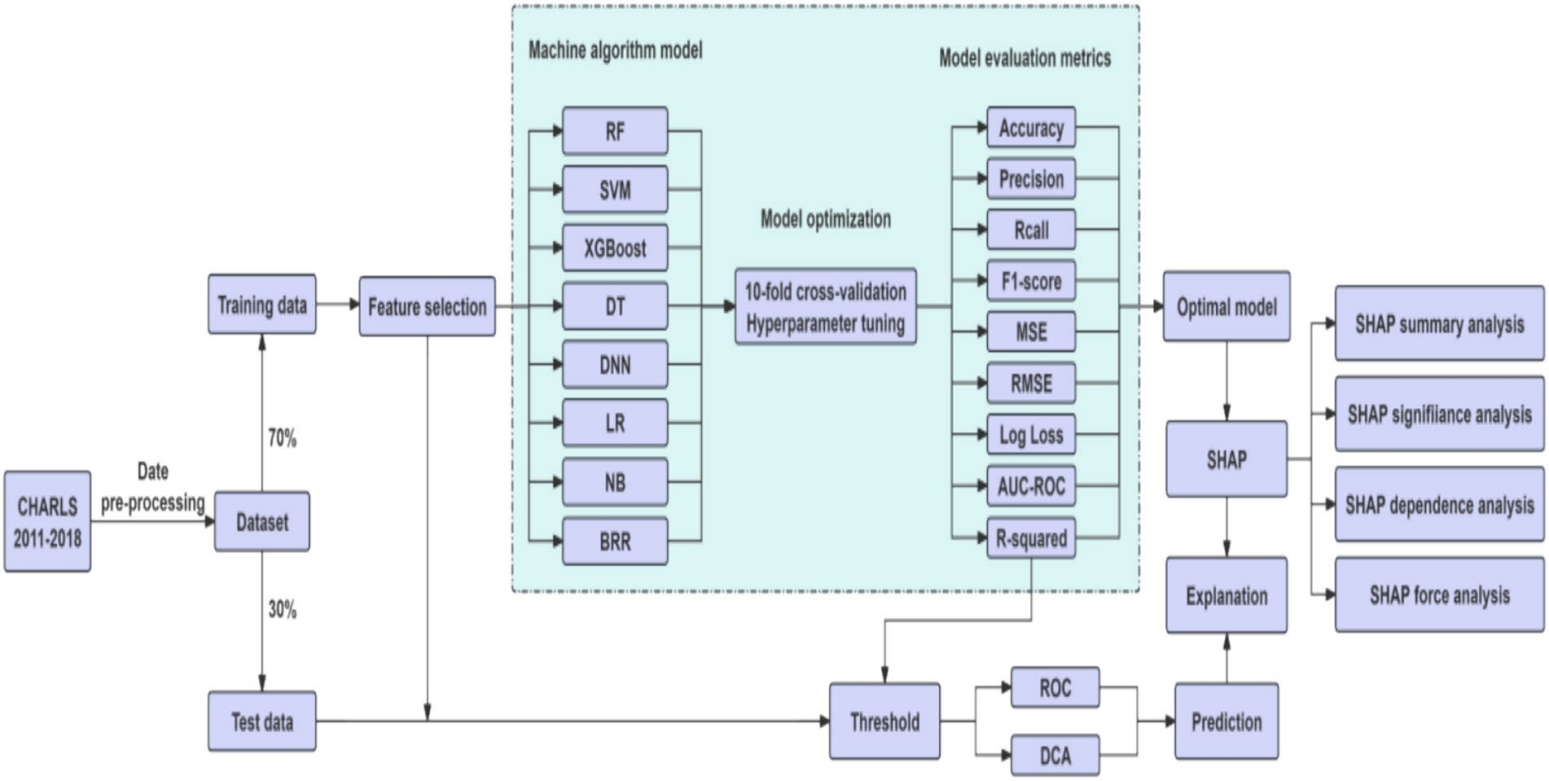
The workflow of study.

The eight algorithms differ significantly in terms of model assumptions, capacity to handle non-linear relationships and feature interactions, interpretability and transparency, sensitivity to data quality and missingness, computational cost and scalability. By comparing these models, our study aimed to identify the most suitable approach for predicting multimorbidity risk among older adult individuals in China. Figure 2 showed the workflow of study.

## 3 Results

### 3.1 Demographic characteristics

A total of 34,755 older adult were included in this study, 8,728 of whom had multimorbidity, and the multimorbidity rate of chronic diseases was 25.1%. Among them, 4,584 are male and 4,144 are female. There were 5,066 people aged 60–69 years old suffering from multimorbidity, 2,892 aged 70–79 years old, and 770 aged over 80 years old. The data were split according to a ratio of 7:3. The training set contained 24,328 cases, of which 6,056 had multimorbidity. The test set contained 10,427 cases, of which 2,672 had multimorbidity. The final demographic characteristics baseline data are as shown in Table 1. Except for hearing problem and medical insurance, there were no statistically significant differences in baseline characteristics between the two groups (*p* > 0.05).

**Table 1:** Characteristics of study population.

Characteristics	Frequency *N*(%)	Multimorbidity *N*(%)	No multimorbidity *N*(%)	*P*-values
Gender				<0.001
Male	17,239(49.6%)	4,584(52.5%)	13,095(50.3%)	
Female	17,516(50.4%)	4,144(47.5%)	12,932(49.7%)	
Age (years)				
60–69	21,483(61.8%)	5,066(58.0%)	16,417(63.1%)	<0.001
70–79	10,255(29.5%)	2,892(33.2%)	7,363(28.3%)	
Over 80	3,017(8.7%)	770(8.8%)	2,247(8.6%)	
Vision problem				
No	4,149(11.9%)	1,352(15.5%)	2,797(10.7%)	<0.001
Yes	30,606(88.1%)	7,376(84.5%)	23,230(89.3%)	
Hearing problem				
No	4,783(13.8%)	1,238(14.2%)	3,545(13.6%)	0.186
Yes	29,972(86.2%)	7,490(85.8%)	22,482(86.4%)	
Disability				
No	28,593(82.3%)	6,592(75.5%)	22,001(84.5%)	<0.001
Yes	6,162(17.7%)	2,136(24.5%)	4,026(15.5%)	
Depression				
No	23,049(66.3%)	4,726(54.1%)	18,323(70.4%)	<0.001
Yes	11,706(33.6%)	4,002(45.9%)	7,704(29.6%)	
Physical pain condition				
No	21,820(62.8%)	5,367(61.5%)	16,453(63.2%)	0.004
Yes	12,935(37.2%)	3,361(38.5%)	9,574(36.8%)	
Self-assessment of health status				
Very good	1787(5.1%)	235(2.7%)	1,552(6.0%)	<0.001
Good	2,826(8.1%)	469(5.4%)	2,357(9.1%)	
In general	18,236(52.5%)	4,358(49.9%)	13,878(53.3%)	
Poor	9,666(27.8%)	2,794(32.0%)	6,872(26.4%)	
Very poor	2,240(6.5%)	872(10.0%)	1,368(5.2%)	
Smoking				
No	18,476(53.2%)	4,865(55.7%)	13,611(52.3%)	<0.001
Yes	16,279(46.8%)	3,863(44.3%)	12,416(47.7%)	
Drinking				
No	23,665(68.1%)	6,320(72.4%)	17,345(66.6%)	<0.001
Yes	11,090(31.9%)	2,408(27.6%)	8,682(33.4%)	
Physical exercise				
No	19,218(55.3%)	5,543(63.5%)	13,675(52.5%)	<0.001
Yes	15,537(44.7%)	3,185(36.5%)	12,352(47.5%)	
Sleep time				
Less than 6 h	12,903(37.1%)	3,539(40.5%)	9,364(36.0%)	<0.001
Between 6–8 h	15,836(45.6%)	4,110(47.1%)	11,726(45.0%)	
Over 8 h	6,016(17.3%)	1,079(12.4%)	4,937(19.0%)	
Nap time				
No	13,880(39.9%)	3,244(37.2%)	10,636(40.9%)	<0.001
Yes	20,875(60.1%)	5,484(62.8%)	15,391(59.1%)	
Social activities				
No	18,105(52.1%)	4,446(50.9%)	13,659(52.5%)	0.013
Yes	16,650(47.9%)	4,282(49.1%)	12,368(47.5%)	
ADL				
No	26,214(75.4%)	4,793(54.9%)	21,421(82.3%)	<0.001
Yes	8,541(24.6%)	3,935(45.1%)	4,606(17.7%)	
IADL				
No	25,684(73.9%)	6,082(69.7%)	19,602(75.3%)	<0.001
Yes	9,071(26.1%)	2,646(30.3%)	6,425(24.7%)	
Marital status				
Married	27,962(80.5%)	6,920(79.3%)	21,042(80.8%)	0.001
Divorce	371(1.0%)	117(1.3%)	254(1%)	
Other	6,422(18.5%)	1,691(19.4%)	4,731(18.2%)	
Residence				
Rural	21,281(61.2%)	4,982(57.1%)	16,299(62.6%)	<0.001
Urban	13,474(38.8%)	3,746(42.9%)	9,728(37.4%)	
Geographical distribution				
West	6,221(17.9%)	2095(24.0%)	4,126(15.9%)	<0.001
Central	15,439(44.4%)	3,673(42.1%)	11,766(45.2%)	
East	13,095(37.7%)	2,960(33.9%)	10,135(38.9%)	
Educational status				
Primary school or below	16,321(47.0%)	5,989(68.6%)	10,332(39.7%)	<0.001
Junior high school	4,180(12.0%)	1,239(14.2%)	2,941(11.3%)	
Senior high school or above	14,254(41.0%)	1,500(17.2%)	12,754(49.0%)	
Per capita annual income (Yuan)				
Less than 5,000	18,769(54.0%)	3,475(39.8%)	15,294(58.8%)	<0.001
5,000–50,000	3,761(10.8%)	996(11.4%)	2,765(10.6%)	
Over 50,000	12,225(35.2%)	4,257(48.8%)	7,968(30.6%)	
Work				
No	20,121(57.9%)	6,181(70.8%)	13,940(53.6%)	<0.001
Yes	14,634(42.1%)	2,547(29.2%)	12,087(46.4%)	
Medical insurance				
No	1,006(2.9%)	236(2.7%)	770(3%)	0.220
Yes	33,749(97.1%)	8,492(97.3%)	25,257(97%)	
Endowment insurance				
No	24,571(70.7%)	6,666(76.4%)	17,905(68.8%)	<0.001
Yes	10,184(29.3%)	2062(23.6%)	8,122(31.2%)	

### 3.2 Feature selection

**Figure 3:**
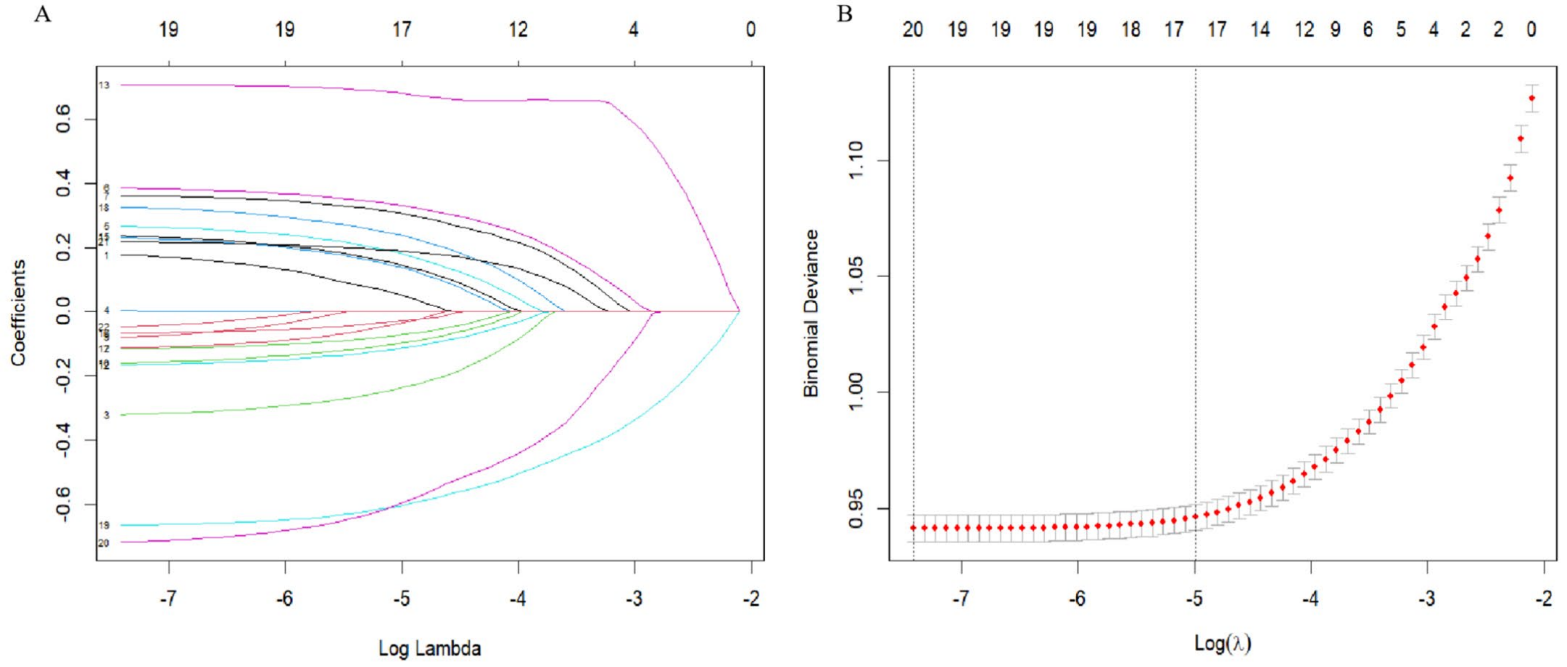
Variable screening based on Lasso regression model. **(A)** The changing characteristics of variable coefficients. **(B)** The selection process of the optimal value of parameter *λ* in the Lasso regression model by cross-validation method.

We used LASSO regression for parameter screening, and the changing characteristics of the coefficients of the variables are shown in Figure 3A. The 10-fold cross-validation method was used for iterative analysis. When *λ* = 0.0068 (Log λ = −4.99), a model with excellent performance and the smallest number of variables was obtained (Figure 3B). Finally, we combined gender, age, vision, disability, self-assessed health status, depression, physical exercise, sleep time at night, nap status, daily activity ability, marital status, geographical distribution, urban and rural distribution, education level, working status, per capita annual income. A total of 17 features were used as predictor variables to develop the machine learning model.

### 3.3 Model evaluation and comparison

Based on the features selected by the LASSO algorithm, we constructed predictive models using eight widely adopted machine learning algorithms: RF, SVM, XGBoost, DT, DNN, LR, NB, and BRR. Model performance was evaluated on both the training and test datasets using a comprehensive set of metrics, including accuracy, precision, recall, F1-score, MSE, RMSE, log loss, AUC-ROC, and R-squared.

**Figure 4:**
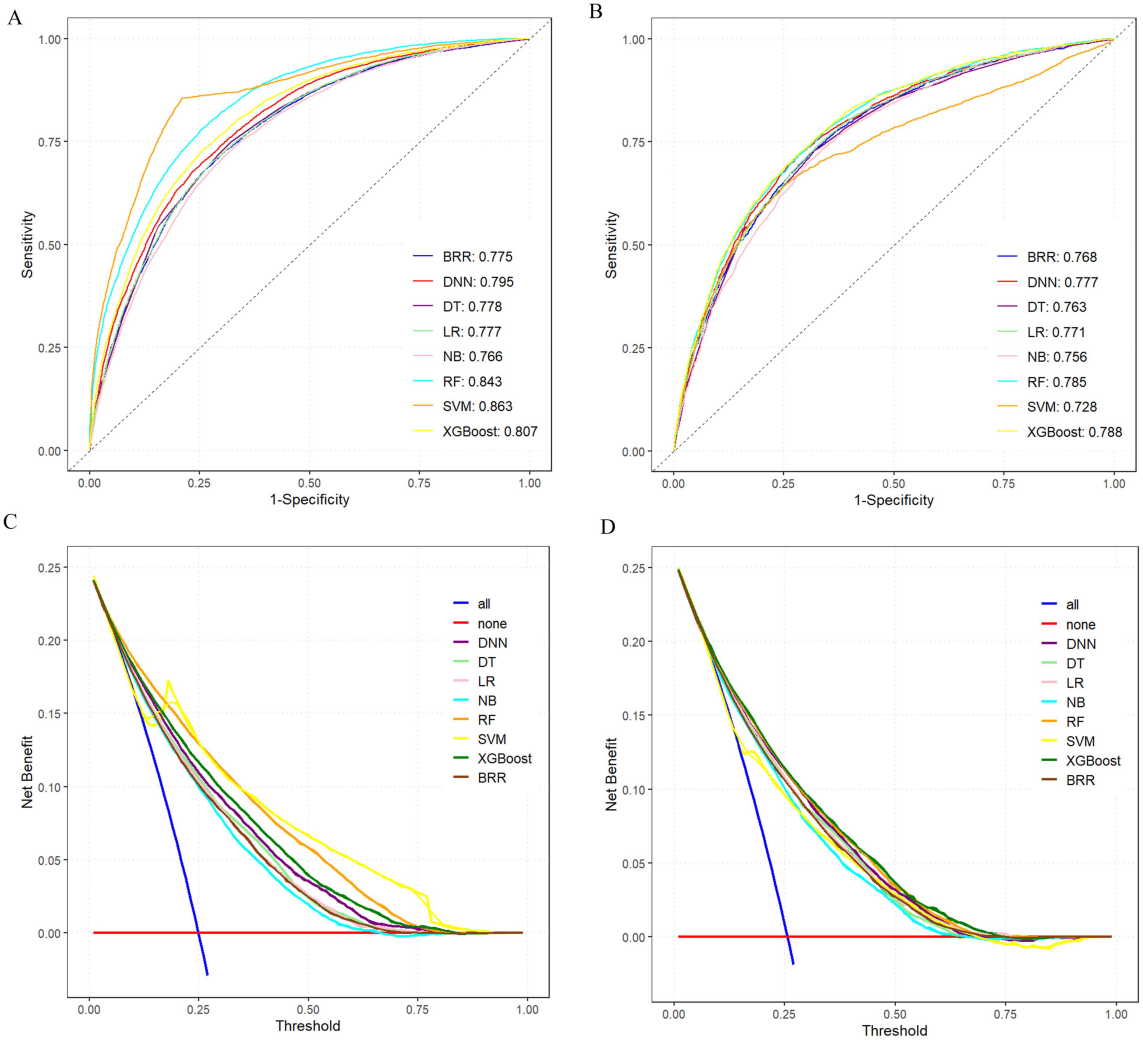
Comprehensive evaluation of machine learning models. **(A)** ROC and AUC of the training set. **(B)** ROC and AUC of the test set. **(C)** DCA of the training set. **(D)** DCA of the test set; where the all curve in the DCA curve represents all situations with intervention. The benefit rate, while the none curve represents the benefit rate in all cases without intervention. The remaining curves represent various models.

Among all models, the XGBoost algorithm demonstrated the most favorable overall performance. It achieved the highest AUC values on both the training set (0.807) and the test set (0.788), indicating superior discriminative ability. In terms of classification metrics, XGBoost maintained competitive accuracy (training: 0.817; test: 0.780), precision (training: 0.805; test: 0.755), recall (training: 0.817; test: 0.775), and F1-score (training: 0.803; test: 0.758), outperforming most other models across these indicators. While the RF and SVM models also exhibited relatively high AUCs (training: 0.843 and 0.863, respectively), their test performance in accuracy and other metrics was slightly lower than that of XGBoost, suggesting potential overfitting or reduced generalizability. Notably, although BRR achieved acceptable accuracy (training: 0.776; test: 0.771) and AUC (training: 0.775; test: 0.768), its recall (training: 0.254; test: 0.255) and F1-score (training: 0.361; test: 0.364) were significantly lower than those of other models, indicating poor sensitivity and suboptimal classification balance, as shown in Figures 4A,B.

To further compare the practical utility of each model, we performed DCA on both the training and test datasets. As shown in Figures 4C,D, XGBoost consistently yielded the highest net benefit across a wide range of threshold probabilities, further supporting its superior clinical applicability. Specific model parameters for each algorithm are detailed in Table 2.

**Table 2:** Evaluates the performance of eight algorithms.

Algorithm	Date set	Accuracy	Precision	Recall	F1-score	MSE	RMSE	Log Loss	AUC-ROC	*R*-squared
RF	Training	0.815	0.805	0.815	0.793	0.191	0.437	0.092	0.843	−0.020
RF	Test	0.779	0.758	0.778	0.752	0.222	0.471	0.107	0.785	−0.165
SVM	Training	0.783	0.761	0.783	0.757	0.180	0.424	0.086	0.863	0.040
SVM	Test	0.775	0.752	0.775	0.748	0.225	0.474	0.108	0.728	−0.181
XGBoost	Training	0.817	0.805	0.817	0.803	0.210	0.458	0.101	0.807	−0.121
XGBoost	Test	0.780	0.755	0.775	0.758	0.220	0.469	0.105	0.788	−0.152
DT	Training	0.775	0.751	0.775	0.751	0.225	0.474	0.108	0.778	−0.203
DT	Test	0.769	0.745	0.769	0.744	0.231	0.481	0.111	0.763	−0.214
DNN	Training	0.746	0.729	0.746	0.735	0.213	0.462	7.368	0.795	−0.141
DNN	Test	0.728	0.713	0.728	0.719	0.224	0.474	7.751	0.777	−0.177
LR	Training	0.777	0.752	0.777	0.748	0.223	0.473	0.107	0.777	−0.195
LR	Test	0.772	0.748	0.772	0.743	0.228	0.478	0.110	0.771	−0.199
NB	Training	0.756	0.752	0.756	0.754	0.229	0.479	0.110	0.766	−0.227
NB	Test	0.748	0.744	0.748	0.746	0.234	0.483	0.112	0.756	−0.226
BRR	Training	0.776	0.623	0.254	0.361	0.224	0.473	0.560	0.775	−0.198
BRR	Test	0.771	0.632	0.255	0.364	0.229	0.478	0.591	0.768	−0.201

Collectively, these findings indicated that the XGBoost model offers the best balance of predictive accuracy, robustness, and clinical utility, and thus represented the optimal choice for risk prediction in this study.

### 3.4 Mode explanation

**Figure 5:**
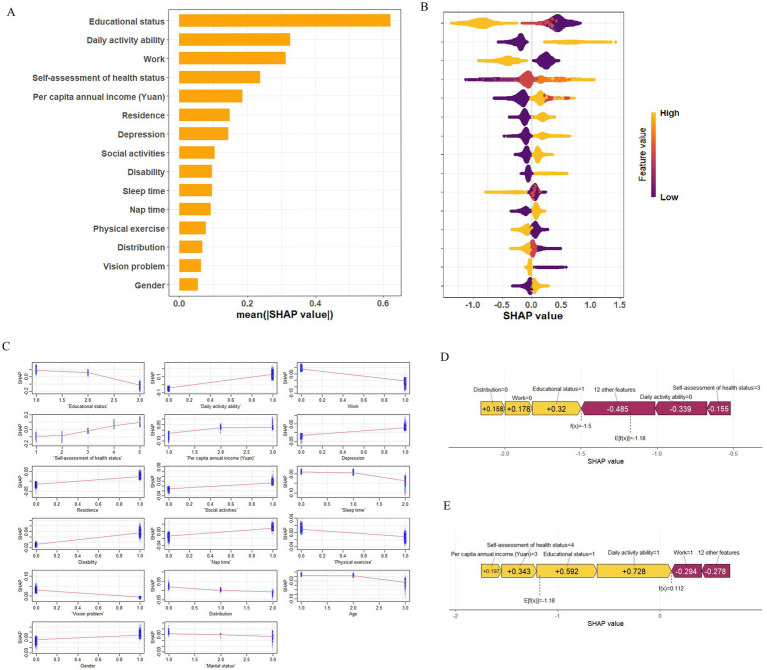
SHAP explains the model. **(A)** Ranking of variable importance based on mean of XGBoost model. **(B)** SHAP honeycomb plot. **(C)** SHAP dependency graph of XGBoost model. **(D)** SHAP prediction for samples without multimorbidity. **(E)** SHAP prediction for multimorbidity samples. Yellow arrowed indicate higher risk of multimorbidity, while red arrows indicated lower risk of multimorbidity. The length of the arrow helped visualize the predicted impact, with longer arrows indicating a more significant effect.

To better understand the relationship between the model and the data, we used SHAP to provide an intuitive interpretation of the XGBoost model to illustrate how these variables affect the risk of multimorbidity in the model. Figure 5A showed the important features in the model, and the ranking of features on the y-axis indicates their importance to the predictive model. The results showed that education level, ADL, working status, self-assessed health status, and per capita annual income were highly correlated with the risk of multimorbidity. Figure 5B illustrated the positive or negative effects of the 15 features affected by XGBoost through SHAP values. The *x*-axis represents the Shapley value, and each feature has positive and negative associations. For example, higher educational level associated negatively to multimorbidity, whereas higher ability to perform daily activities associated positively to multimorbidity. The SHAP dependency diagram can help understand the association of a single feature on the output of the XGBoost prediction model (Figure 5C). For example, lower self-evaluation of health has a negative association to multimorbidity, while having a job has a positive association to multimorbidity. In addition, we provide two typical examples, one predicting participants without multimorbidity (Figure 5D) and the other predicting participants with multimorbidity (Figure 5E). In the prediction of the risk of multimorbidity without chronic disease, education, ADL, work status, and other factors were the main associations, while in the prediction of the risk of multimorbidity with chronic disease, ADL, education status, self-assessment health status, and work status were the main associations. All yellow bars on the left side of the figure represent features that had a positive association deviating from the base value, while red bars on the right side represented features that had a negative association deviating from the base value. The length of the bar chart showed the association of the features, further demonstrating the interpretability of the model.

The results showed that for the age-based subgroups, the top five influencing factors were highly consistent between the 60–69 and 70–79 age groups. These included education status, ADL, work status, self-assessment health status, and per capita annual income, with test set AUCs of 0.796 and 0.790, respectively. In contrast, for individuals aged ≥80 years, the top factors were education status, per capita annual income, residence, depression, and ADL, yielding a slightly lower AUC of 0.728 (Supplementary Figures S1–S4). This suggested a rising influence of psychological and geographic factors in the oldest age group.

For the sex-based subgroups, the key factors for males were education status, work status, ADL, self-assessment health status, and per capita annual income (AUC = 0.790). For females, the main factors were education status, ADL, self-assessment health status, work status, and residence (AUC = 0.792) (Supplementary Figures S5, S6).

## 4 Discussion

This study integrated multiple machine learning algorithms with interpretable analysis techniques (SHAP) to construct a predictive model for multimorbidity risk among the older adult in China. The results indicated that the XGBoost model outperformed others in predictive performance.

SHAP analysis further highlighted the strong association of socioeconomic status (e.g., education level, income, work status) and lifestyle (e.g., ADL, self-assessment health status) in the formation of multimorbidity. These findings aligned closely with theories of social stratification and health lifestyles, supporting the significant association of socioeconomic factors on health outcomes and revealing significant behavioral differences across social strata.

Previous research has shown that individuals with lower socioeconomic status tend to bear a heavier disease burden, face higher rates of chronic illness, and have worse health outcomes (33–35). In China, this issue is particularly complex due to the dual urban–rural structure, regional disparities in development, and differences in social welfare systems, all of which exacerbate health inequality (36). Studies have demonstrated that rural residents are experiencing significantly higher growth rates of obesity, hypertension, and diabetes compared to urban residents (37, 38), largely due to lower education levels, poor health awareness, and limited access to medical resources (33–35). Our findings are consistent, showing that higher education levels are strongly associated with reduced multimorbidity risk.

Education, representing cultural capital, is associated with enhanced health literacy and self-management capabilities (39, 40). The high multimorbidity rate among rural older adult further confirmed the existence of a “health gradient,” where social class shaped health trajectories through access to economic resources, occupational conditions, and health behavior choices (41, 42). Additionally, this study found that reduced ADL and poor self-assessment health status were significant predictors of multimorbidity, indicating that maintaining physical function is associated with a lower risk of delaying the onset of multiple chronic diseases. The U. S. NHATS study found significant associations between multimorbidity and IADL ability, where individuals with five or more chronic conditions exhibited substantial limitations in daily functioning (43). Self-assessment health status, as a multidimensional indicator, reflected not only physical functioning but also psychological well-being and social support (44). In our study, poorer self-assessment health status was significantly associated with a higher risk of multimorbidity, supporting findings from the Italian centenarian study (45), which demonstrated that subjective health perception is driven by emotional well-being and functional ability, while indirectly influenced by socioeconomic status. Thus, self-assessment health status could serve as a practical screening tool for identifying high-risk older adult individuals, especially in resource-limited rural areas (46, 47). Individuals aged ≤79 were mainly influenced by economic and behavioral factors, while those aged ≥80 showed stronger associations with psychological health and geographic location (48). Males were more influenced by occupational and economic security, whereas females appeared more sensitive to living environment and perceived health. These subgroup differences suggested potential avenues for more tailored interventions. The prominence of economic and functional factors (education, work, ADL, income, self-rated health) in the younger older adult (60–79 years) underscored the importance of socioeconomic support and maintaining functional capacity during the earlier stages of aging to potentially mitigate multimorbidity development (49–51). In contrast, the increased relative importance of psychological well-being (depression) and geographic location (residence) among the oldest old (≥80 years) (52–55) highlighted the need to integrate mental health support and address potential barriers related to location (e.g., access to care, social isolation) in interventions. The finding that males appeared more sensitive to occupational and economic security factors, while females were more sensitive to living environment and perceived health (56–58), suggested that gender-specific approaches considering these distinct sensitivities might enhance intervention effectiveness (59). For instance, programs for older adult men might benefit from components addressing financial security or retirement transition, while programs for women might place greater emphasis on improving living conditions and self-efficacy in health management.

Using the LASSO method, this study identified 17 key variables encompassing demographic characteristics, socioeconomic factors, and lifestyle behaviors, underscoring their substantial predictive value and clinical relevance in identifying individuals at risk for multimorbidity (60, 61). The predictive performance of the eight machine learning algorithms evaluated in this study was generally robust, with AUC values ranging from 0.728 to 0.788. Among them, the XGBoost model consistently achieved the highest AUC in both the training and test sets, demonstrating superior discrimination ability. To assess the clinical applicability of each model, we conducted DCA, which revealed that XGBoost provided the greatest net benefit across a wide range of threshold probabilities, reinforcing its potential as a practical tool for early identification of high-risk individuals (62).

From a methodological perspective, non-linear models generally outperformed linear models across multiple evaluation metrics, including accuracy, F1-score, RMSE, and log loss. In particular, XGBoost, which integrates a gradient boosting framework with regularization techniques, demonstrated enhanced ability to model complex, non-linear interactions among predictors. This advantage is especially important in the context of multimorbidity, where the interplay between socioeconomic status, demographic profiles, and lifestyle risk factors is often intricate and multidimensional (63). In contrast, linear models such as logistic regression and Bayesian ridge regression, while offering interpretability and computational efficiency, are inherently limited in their capacity to capture non-linear associations. Consequently, they may underestimate the effects of key predictors or fail to detect higher-order interactions that are clinically meaningful (64). Taken together, these findings suggest that non-linear ensemble models, particularly XGBoost, provide a more accurate and clinically useful approach for multimorbidity risk prediction in population-based datasets. Future studies should explore the integration of model explainability techniques, such as SHAP values, to enhance interpretability without compromising predictive performance, thereby facilitating real-world implementation in preventive healthcare settings.

SHAP analysis visually demonstrated the impact of each feature on prediction outcomes. For instance, higher education levels were associated with negative SHAP values, indicating a protective effect against multimorbidity. Other key variables, ADL, work status, self-assessment health status, and per capita annual income also had significant impacts on predictions. SHAP summary and dependence plots further illustrated how these features contributed to individual risk predictions and how the presence of chronic diseases modulated feature importance, thereby enhancing model interpretability.

These findings not only supported the optimization of predictive models but also underscored the importance of addressing the structural roots of medical issues. Policymakers should increase investment in health education, particularly for low-SES groups, and work to improve the equitable distribution of medical resources. Based on the key features identified, early risk assessment tools for multimorbidity could be developed to optimize intervention strategies and clinical trial design, improving resource efficiency and targeting.

Despite the significance of these findings, this study has several limitations. Firstly, this study is the reliance solely on Chinese data from the CHARLS cohort for model development and validation. While CHARLS provides a nationally representative sample of the Chinese older adult population, the unique socioeconomic transitions, healthcare system structure of China may not directly translate to populations in other countries. External validation using datasets from diverse international cohorts is essential to assess the generalizability and potential applicability of our findings and the XGBoost model. Secondly, this study was a cross-sectional design. Although our analysis identified robust associations between socio-economic factors, lifestyle variables and the risk of chronic disease comorbidity, it was not possible to make causal inferences. Future longitudinal studies or intervention studies are needed to determine the causal relationship.

## 5 Conclusion

By applying machine learning techniques alongside interpretability tools (SHAP) to a large, nationally representative dataset, this study robustly identified key socioeconomic, functional, and lifestyle factors associated with multimorbidity among older adults in China. Integrating machine learning with sociological theory, the study constructed an interpretable model that highlighted variables such as education level, ADL, work status, self-rated health, and income serve as effective predictive factors and reflect deep associations linked to the broader social determinants of health. These findings offer both theoretical insights and practical implications for understanding the underlying mechanisms of multimorbidity, enhancing model interpretability, and informing targeted public health strategies. Future studies incorporating external validation datasets from diverse populations are needed to confirm the applicability of our models.

## Data Availability

Publicly available datasets were analyzed in this study. This data can be found: https://charls.pku.edu.cn/en/.

## Footnotes

^1^ https://charls.charlsdata.com/pages/data/111/zh-cn.html
