# Supplementary material for: Socioeconomic status and lifestyle as factors of multimorbidity among older adults in China: results from the China Health and Retirement Longitudinal Survey
Source: Front Public Health. 2025 Jul 30;13:1586091. doi: 10.3389/fpubh.2025.1586091 (PMC12343554; doi:10.3389/fpubh.2025.1586091)
Supplement: Supplementary file 1 [file Supplementary_file_1.docx]

Supplementary Figures

Fig.S1 Subgroup analysis results of machine learning models. (A) ROC and AUC of the training set; (B) ROC and AUC of the test set;

Fig.S2 Subgroup analysis results for the age group of 60-69 years old based on the XGboost model;

Fig.S3 Subgroup analysis results for the age group of 70-79 years old based on the XGboost model;

Fig.S4 Subgroup analysis results for the age group of >80 years old based on the XGboost model;

Fig.S5 Subgroup analysis results for the gender group in male based on the XGboost model;

Fig.S6 Subgroup analysis results for the gender group in female based on the XGboost model.


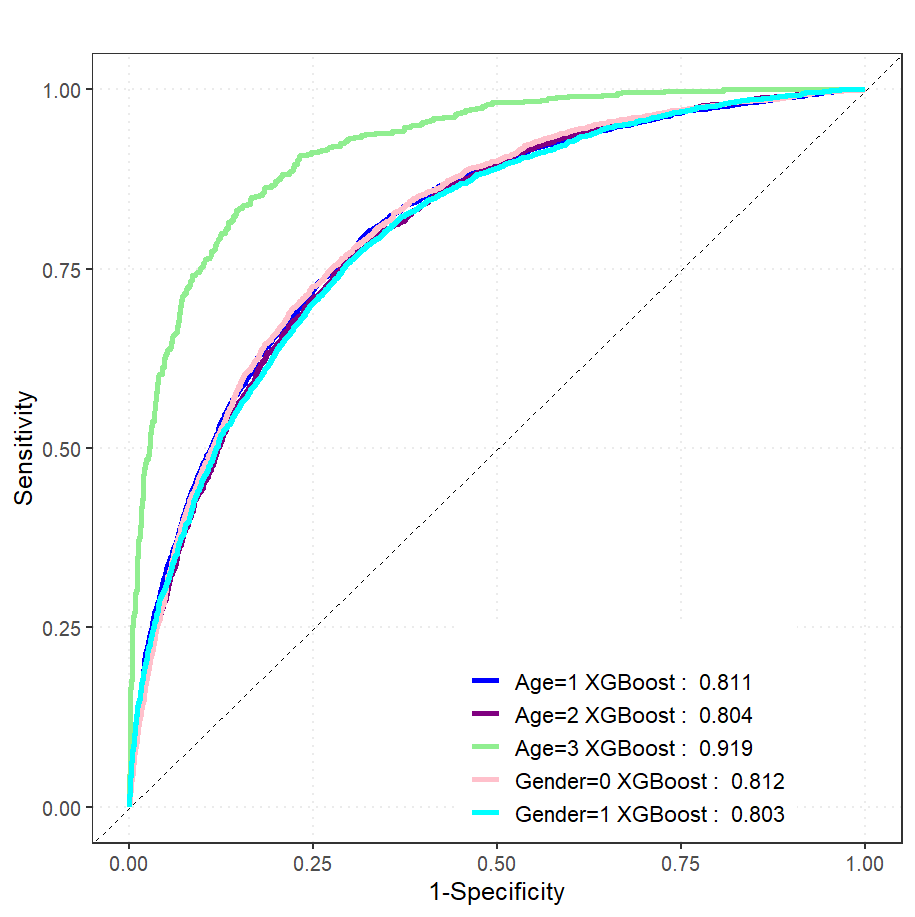


A


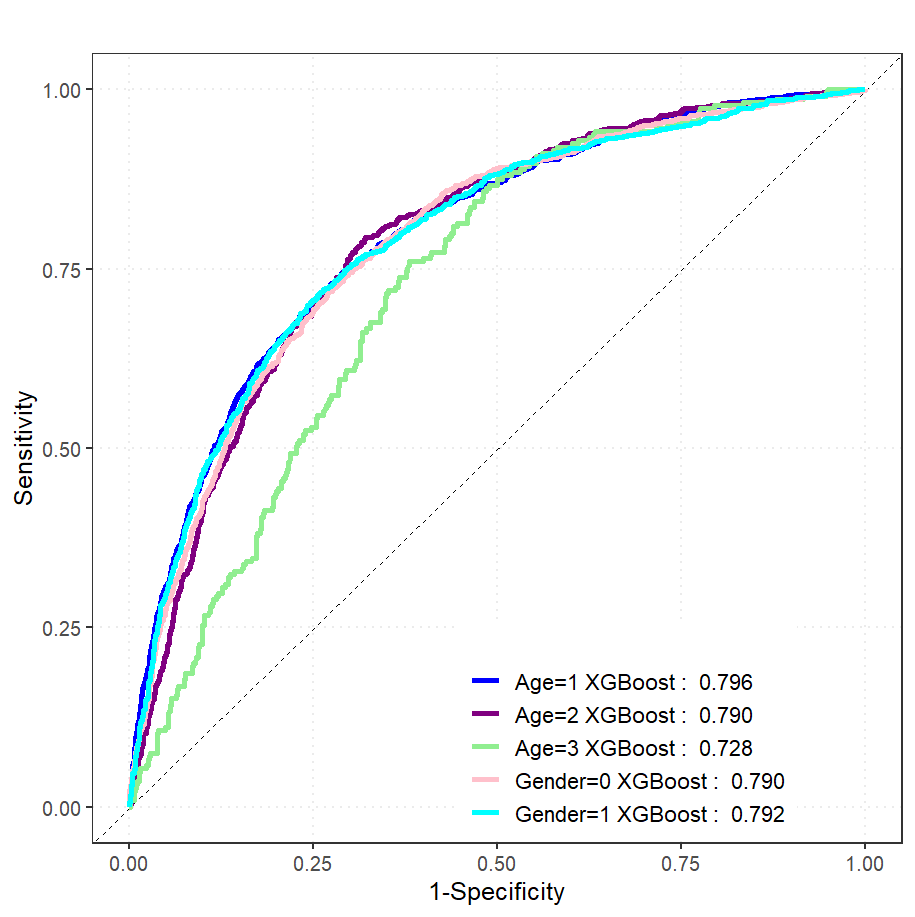


B

Fig.S1 Subgroup analysis results of machine learning models. (A) ROC and AUC of the training set; (B) ROC and AUC of the test set;


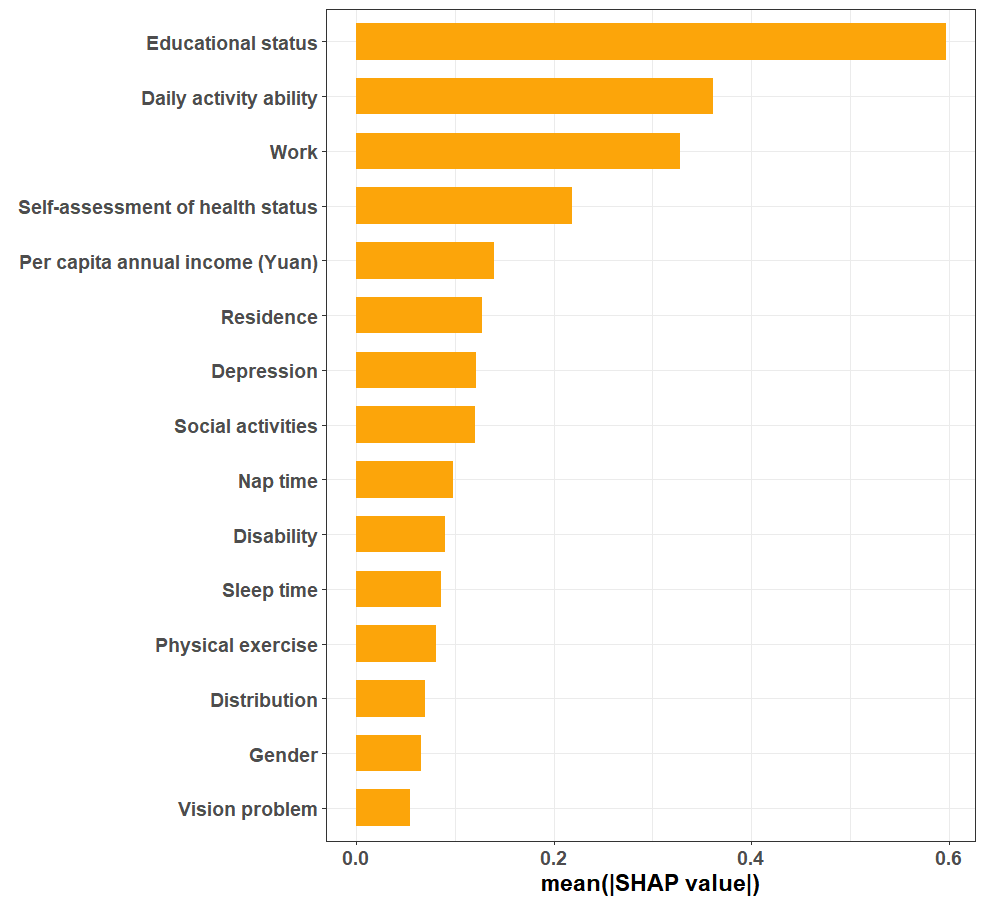

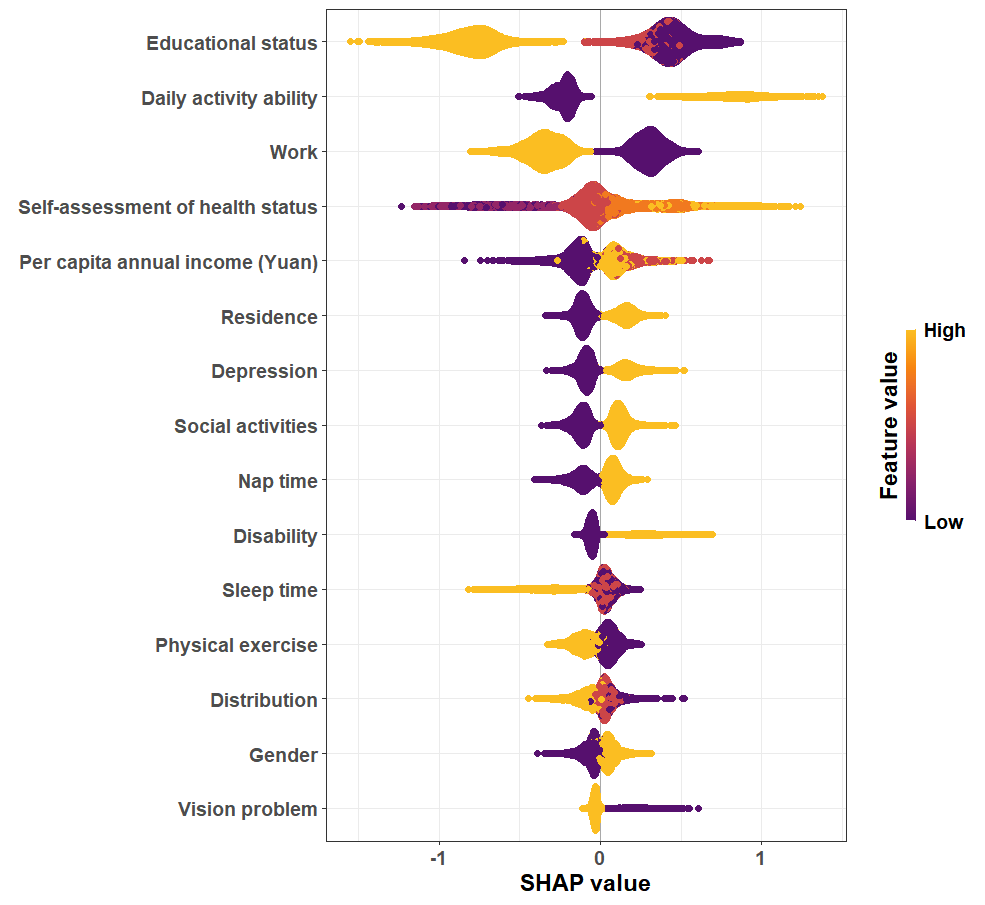

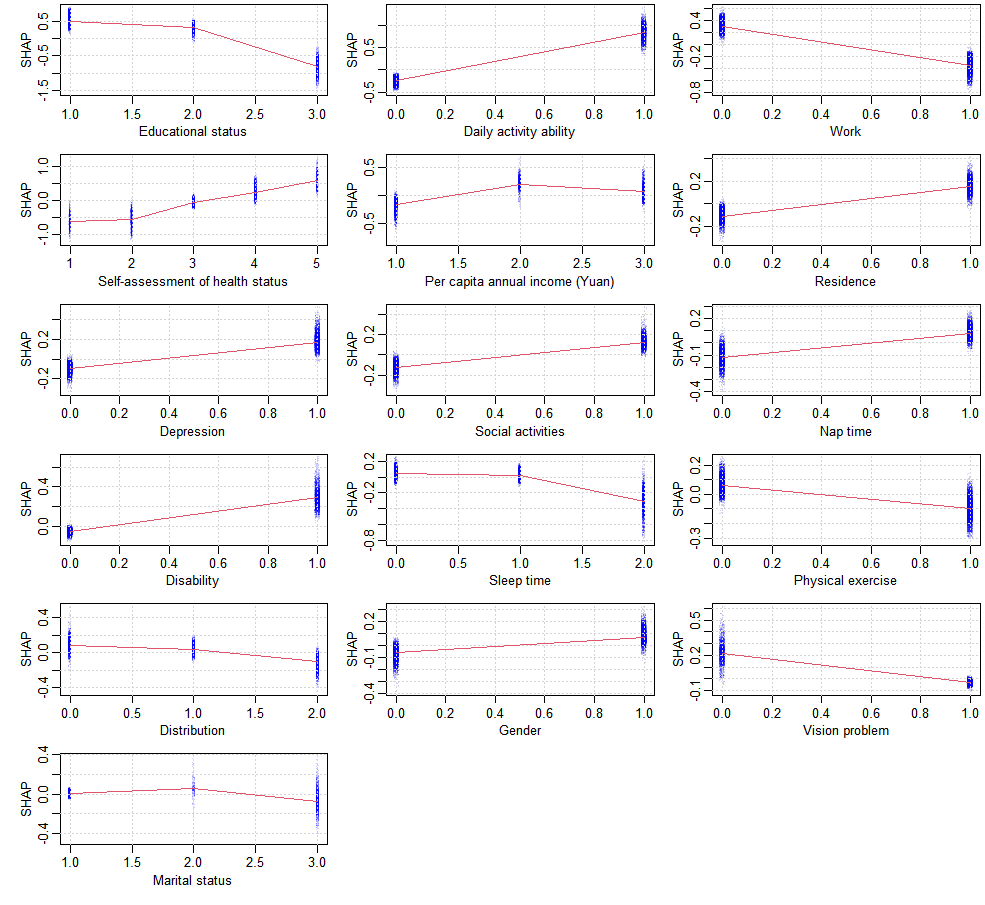

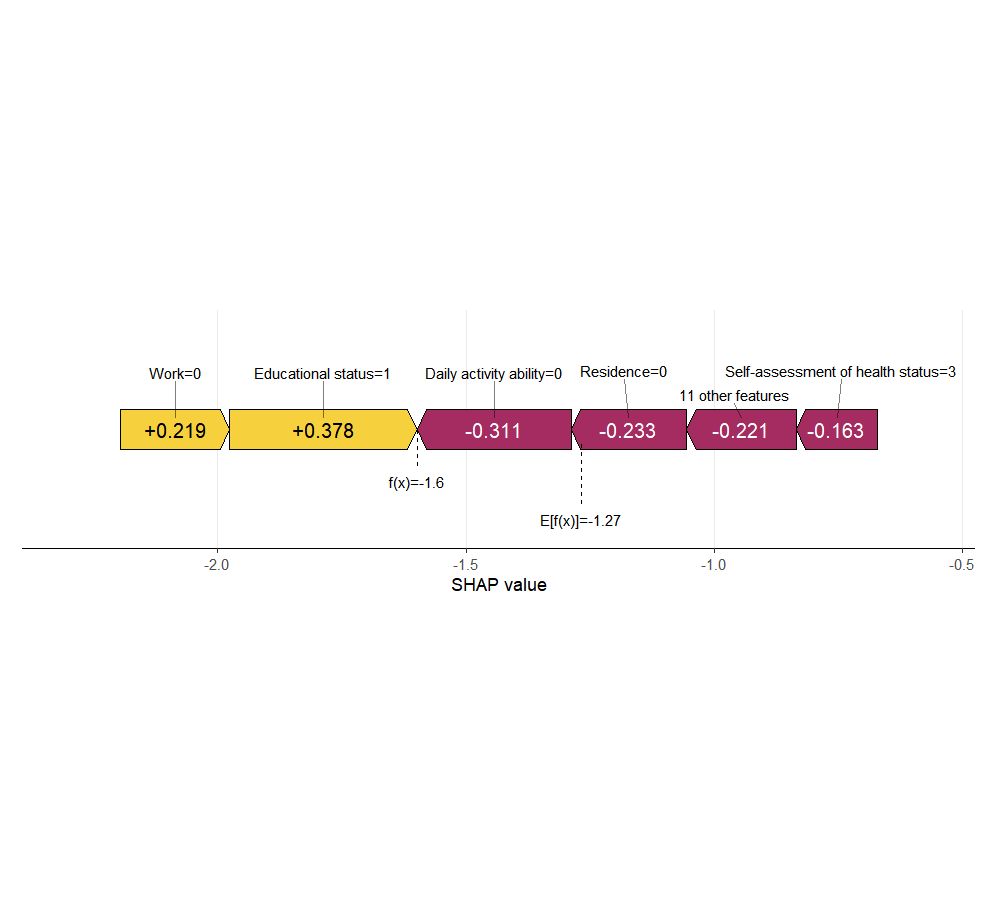

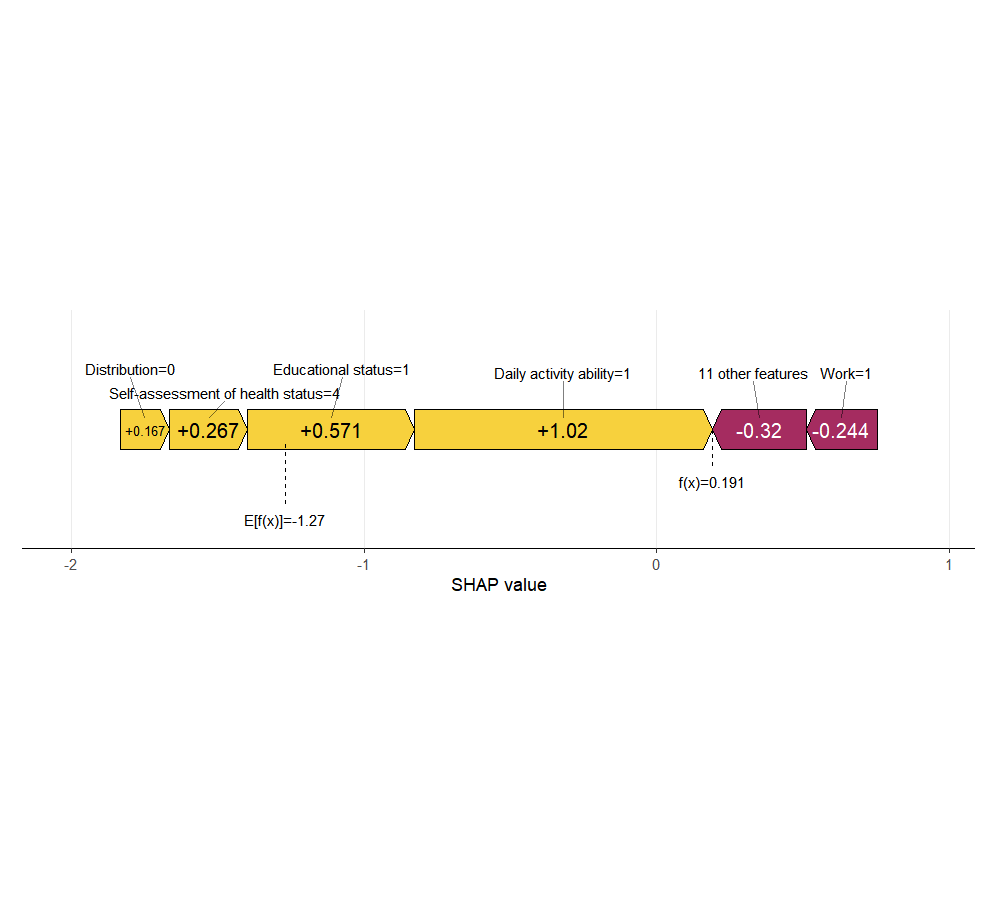


Fig.S2 Subgroup analysis results for the age group of 60-69 years old based on the XGboost model


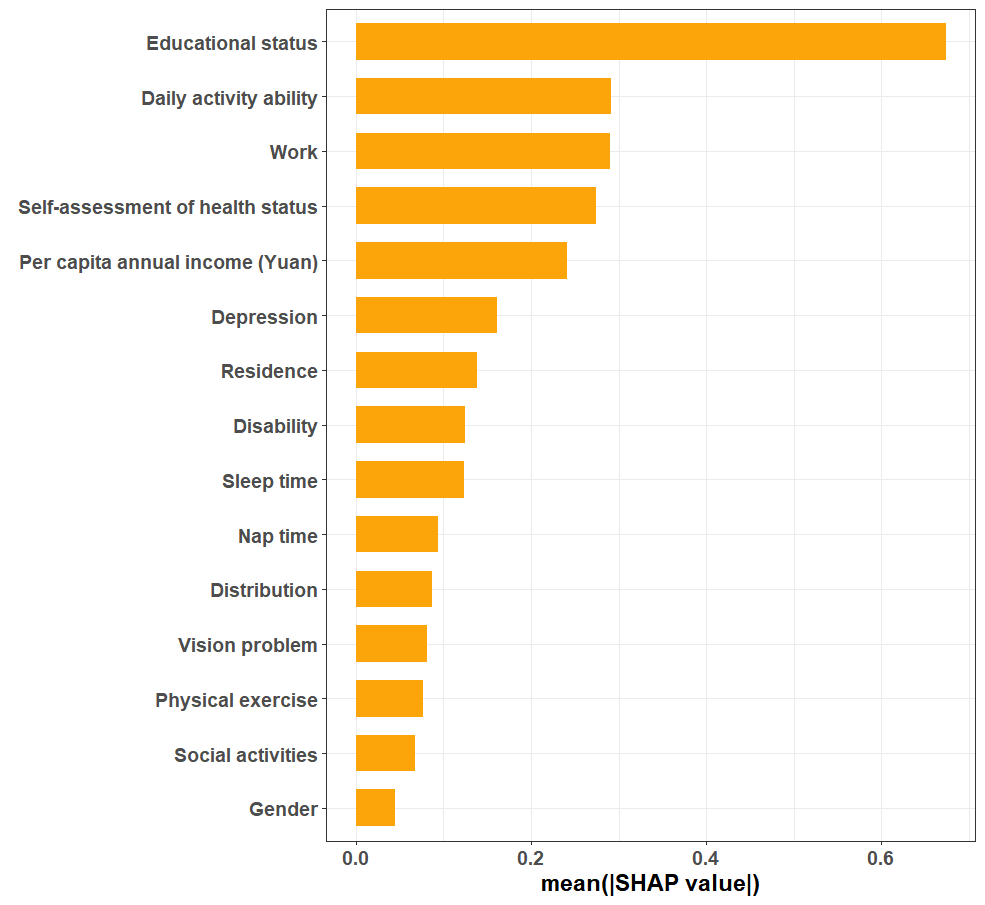

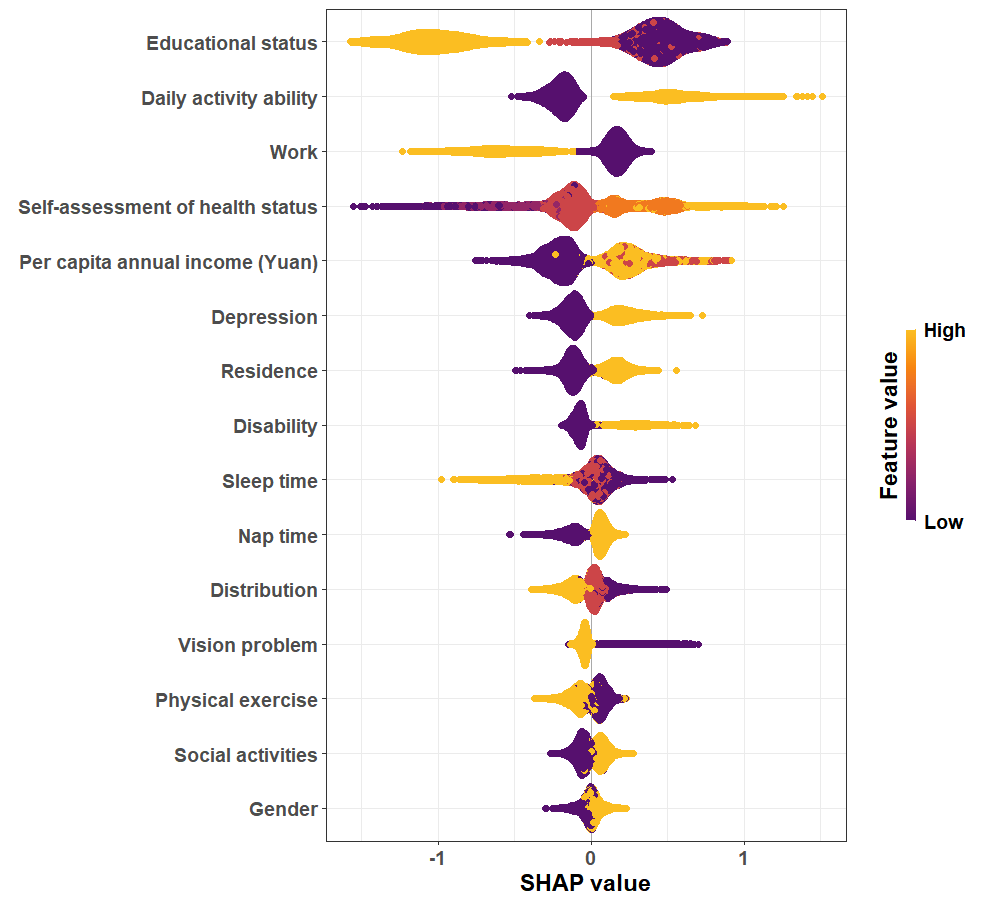

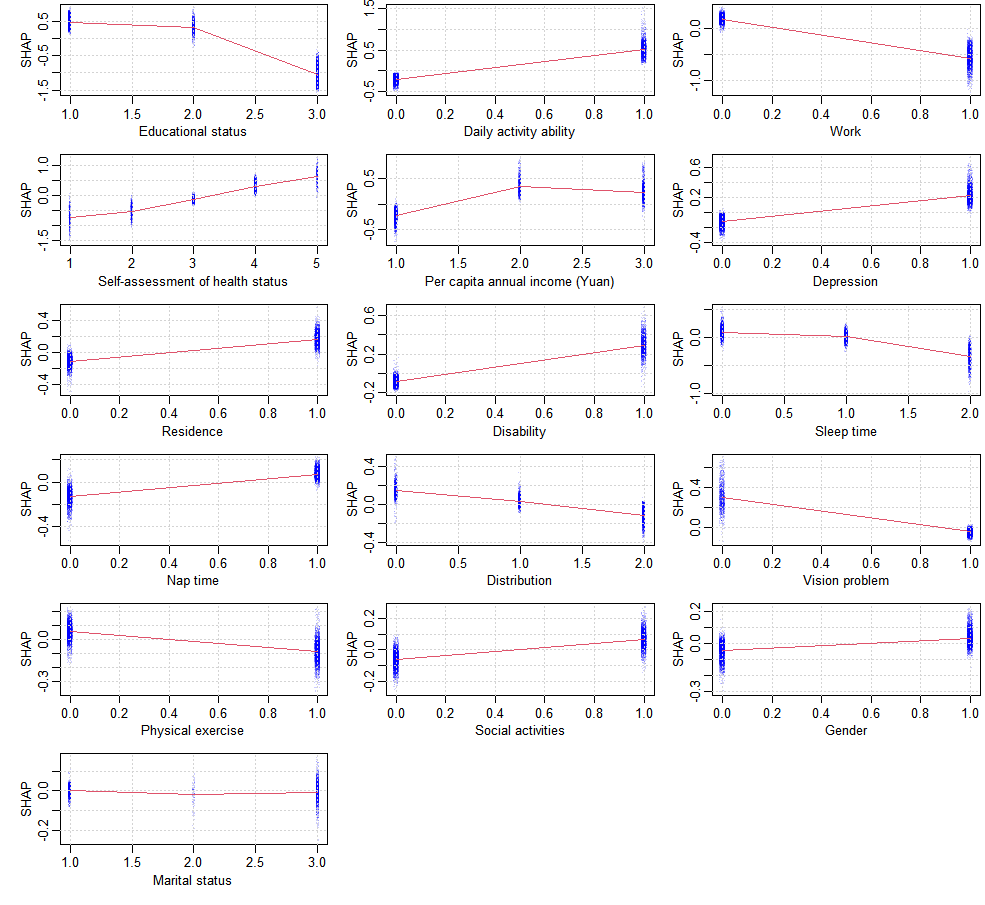

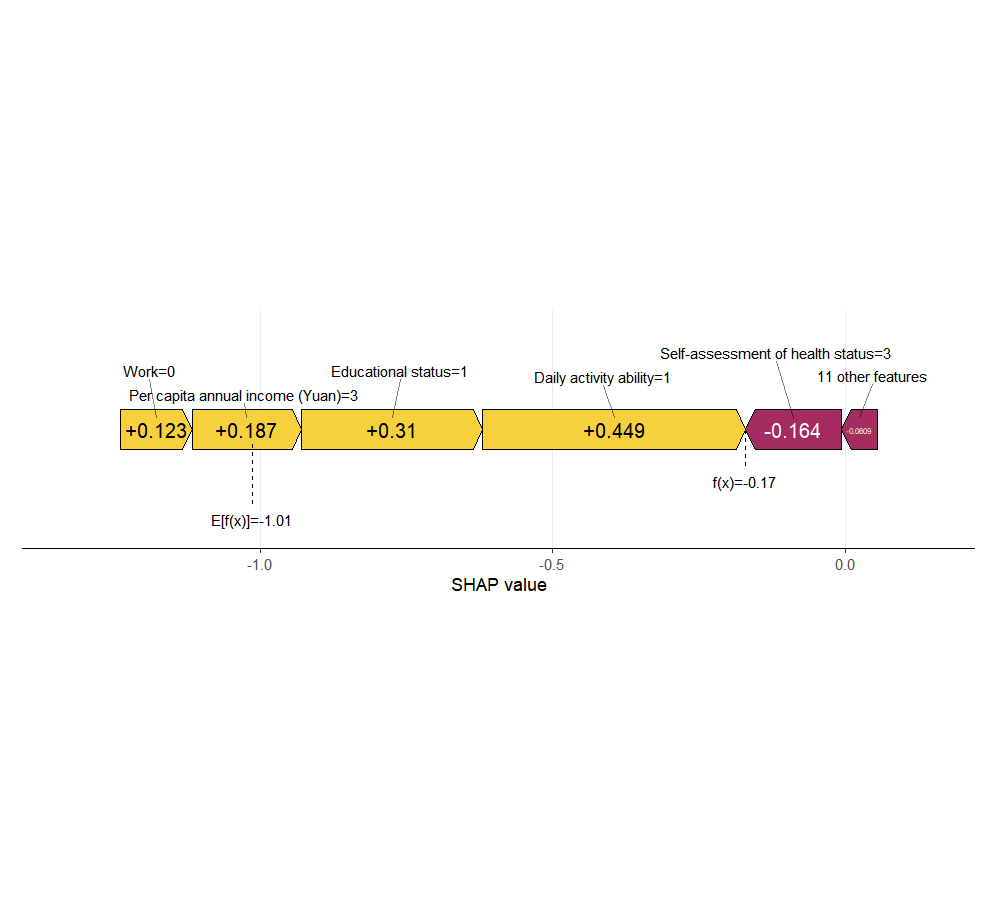

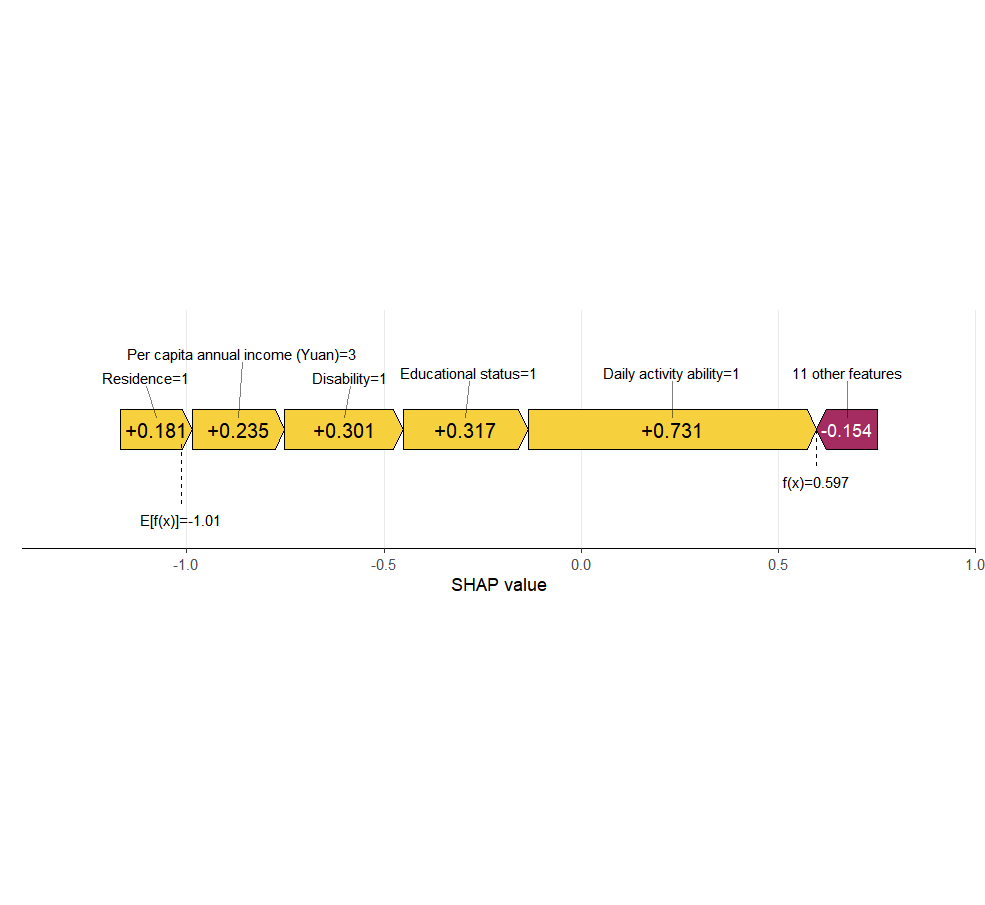


Fig.S3 Subgroup analysis results for the age group of 70-79 years old based on the XGboost model


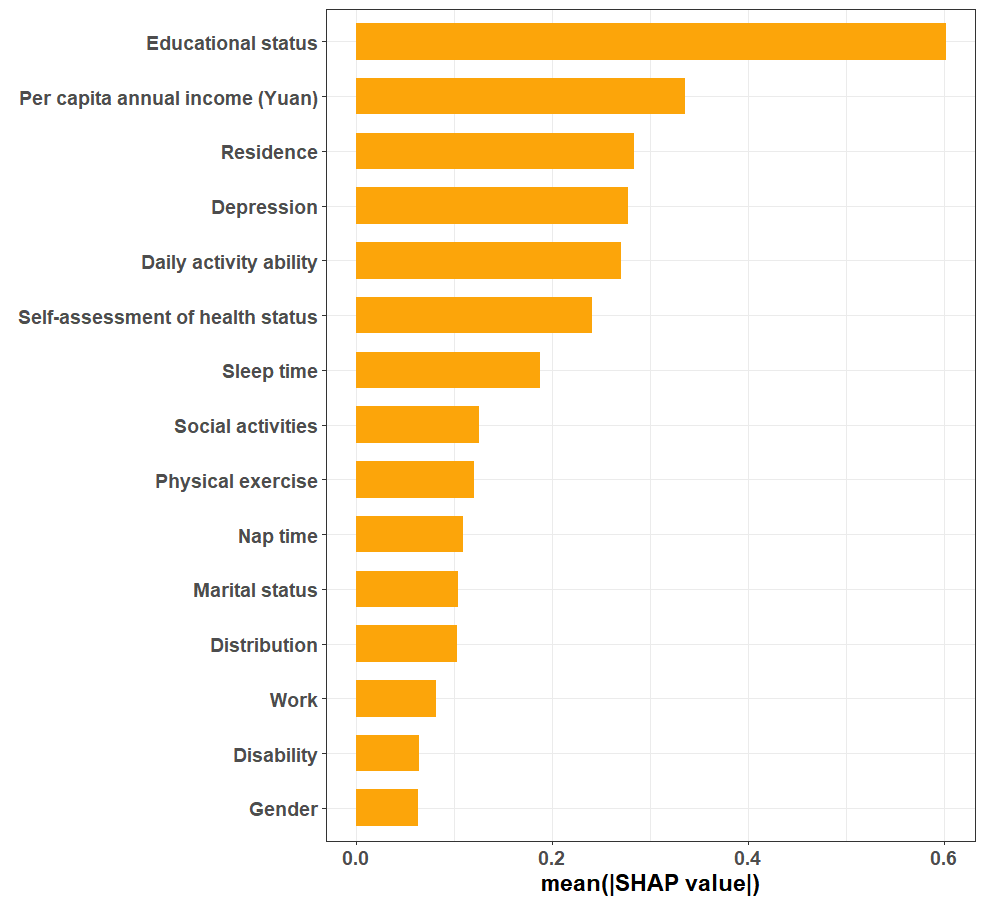

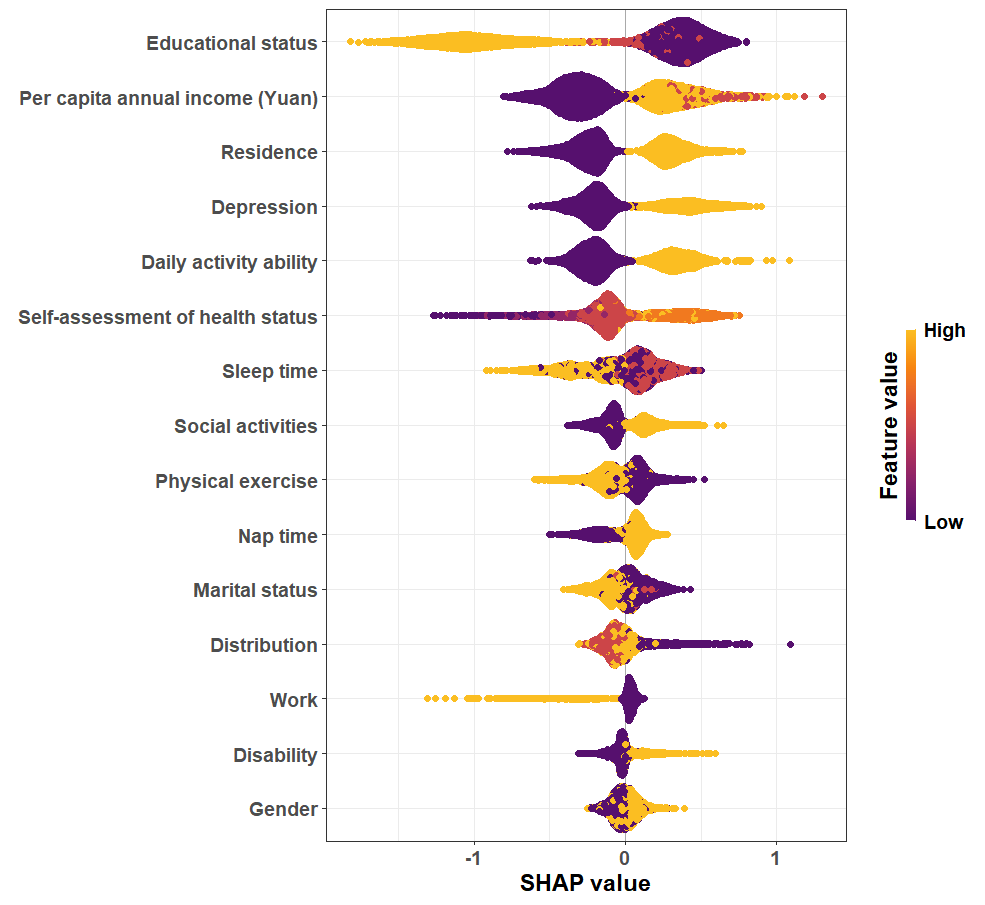

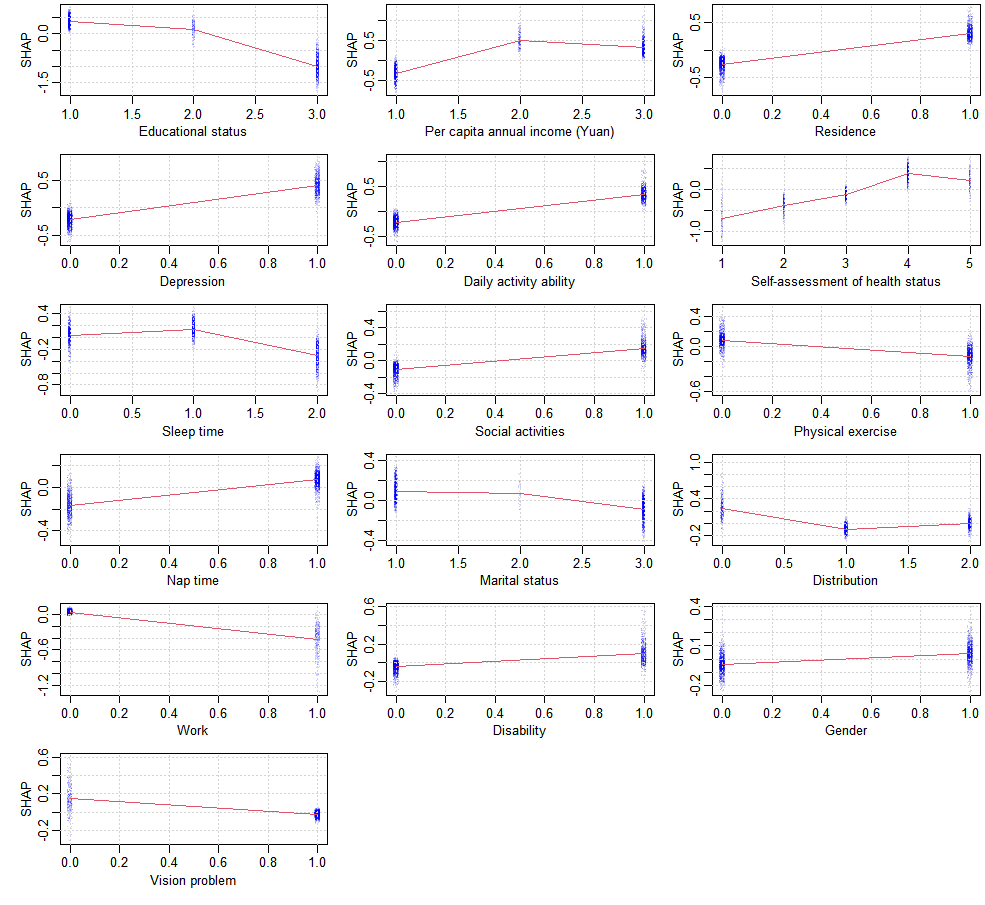

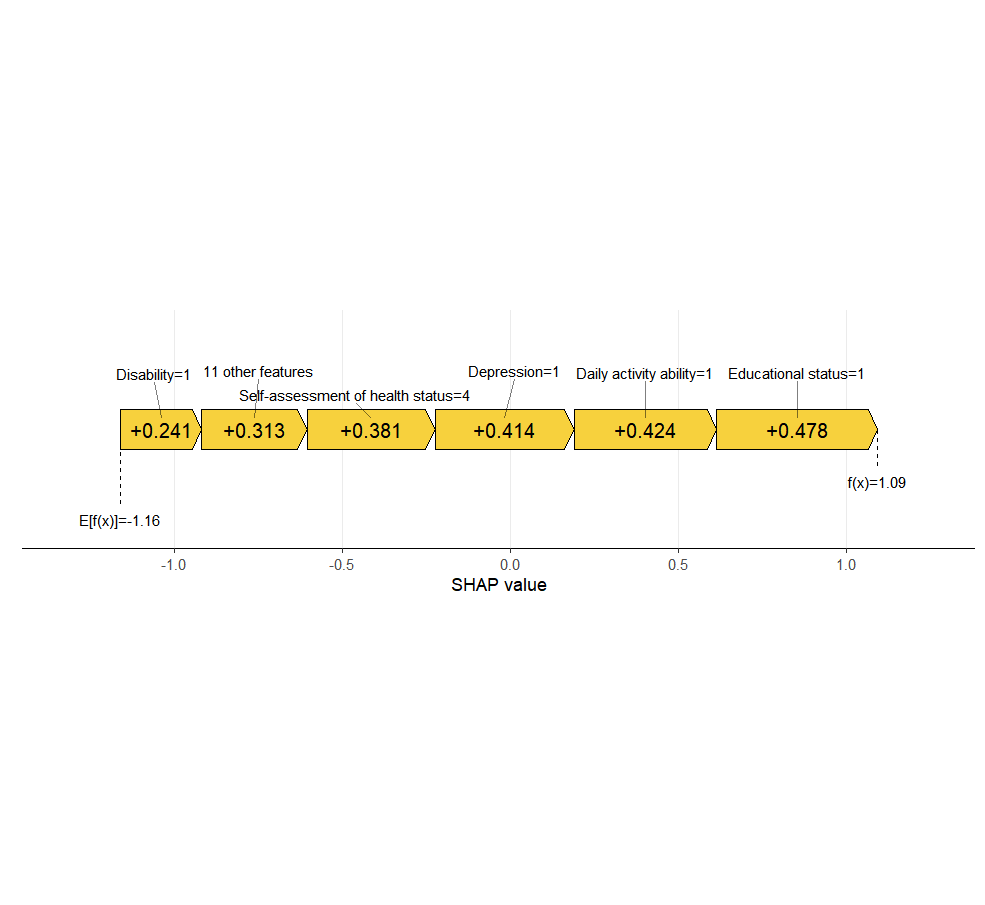

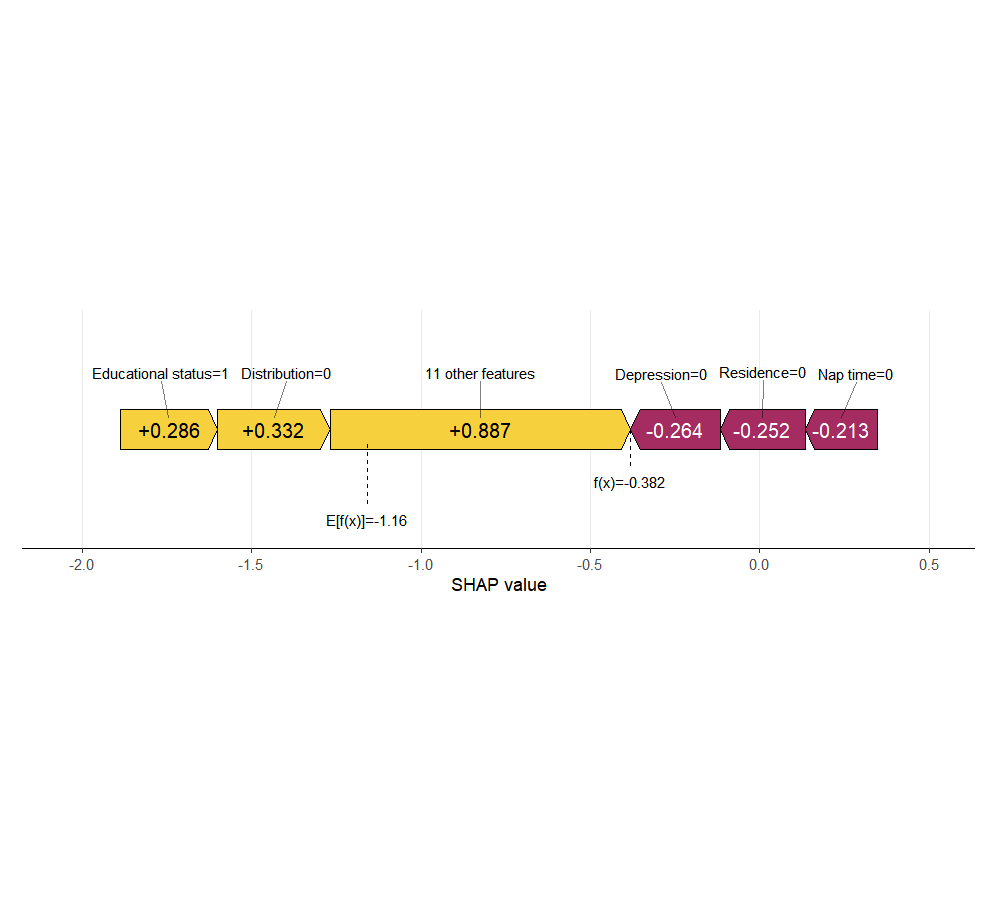


Fig.S4 Subgroup analysis results for the age group of >80 years old based on the XGboost model


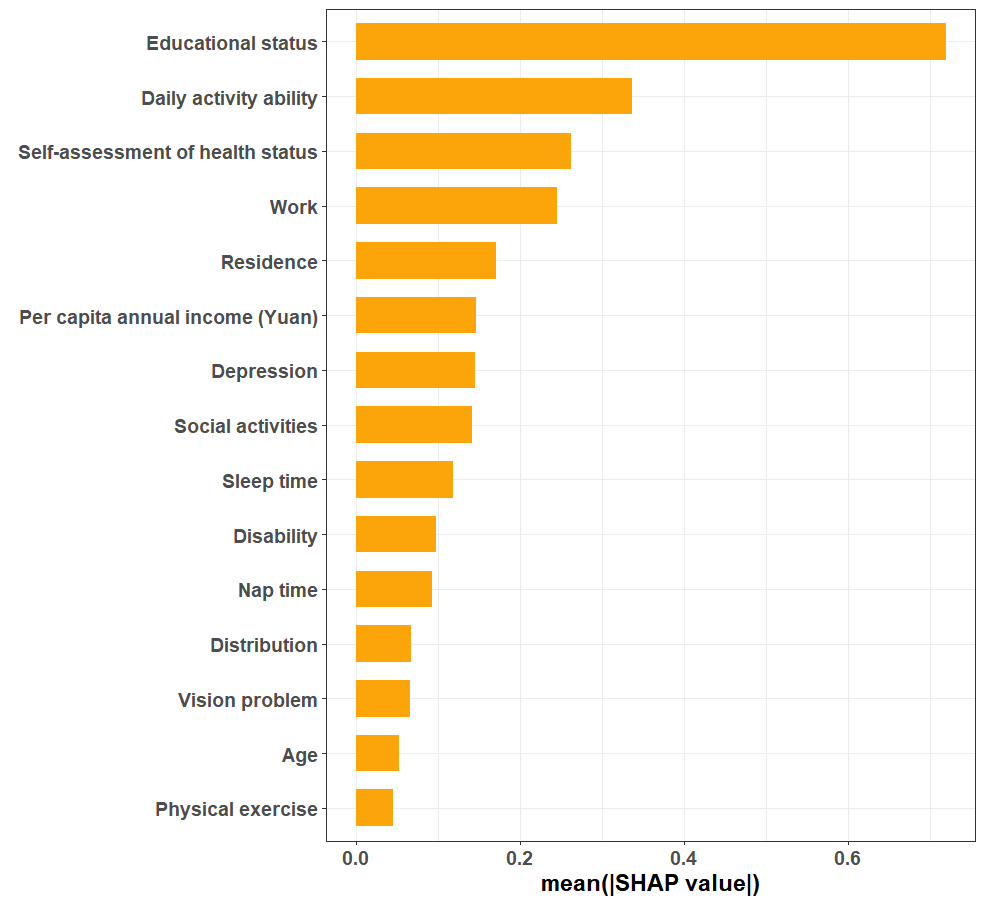

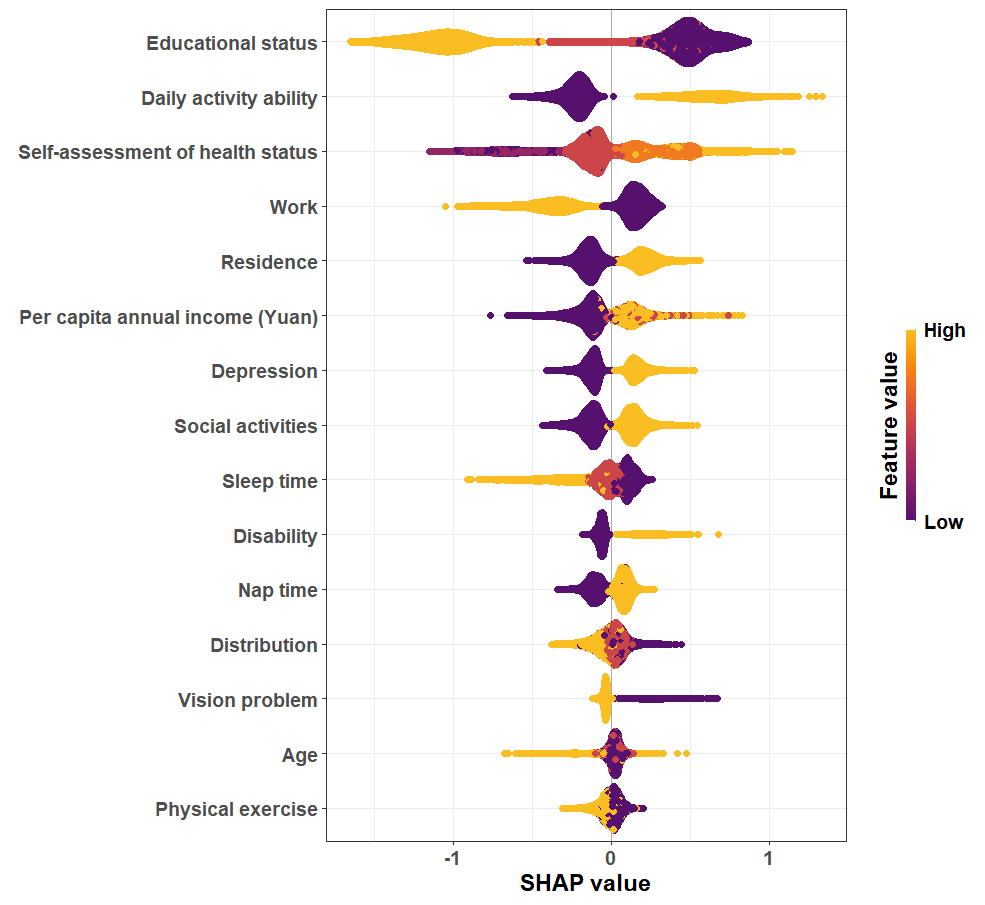

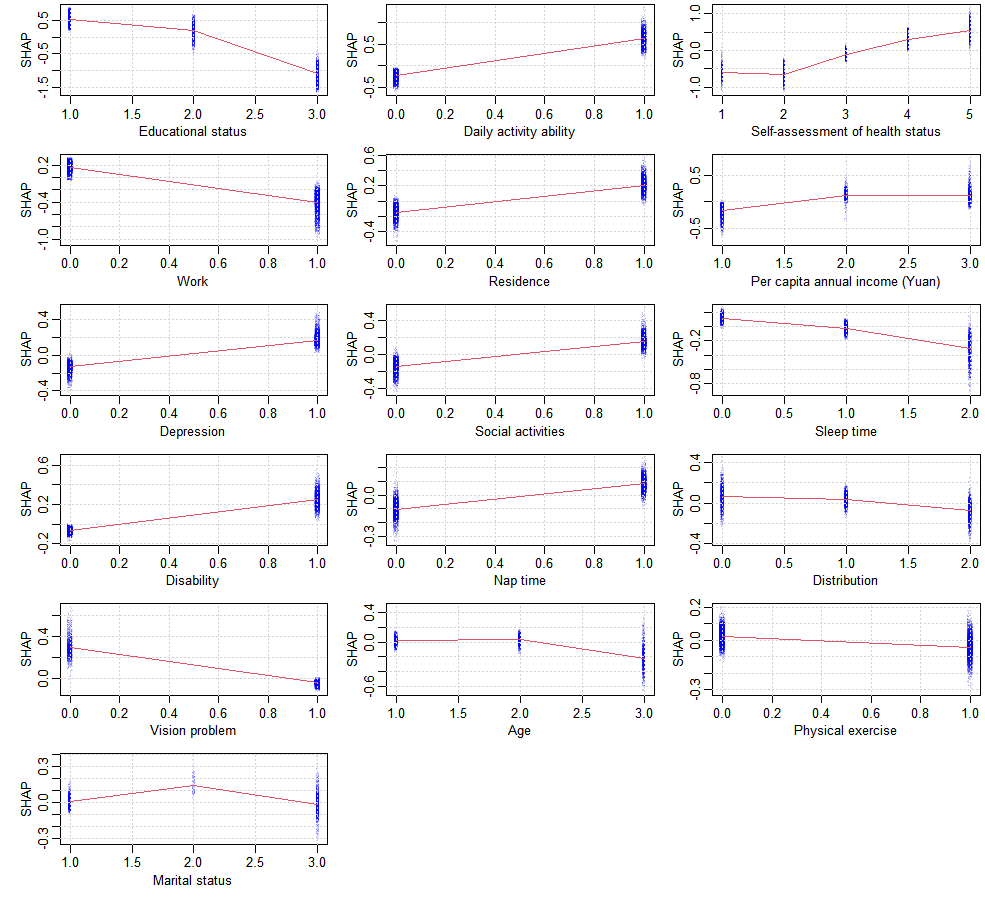

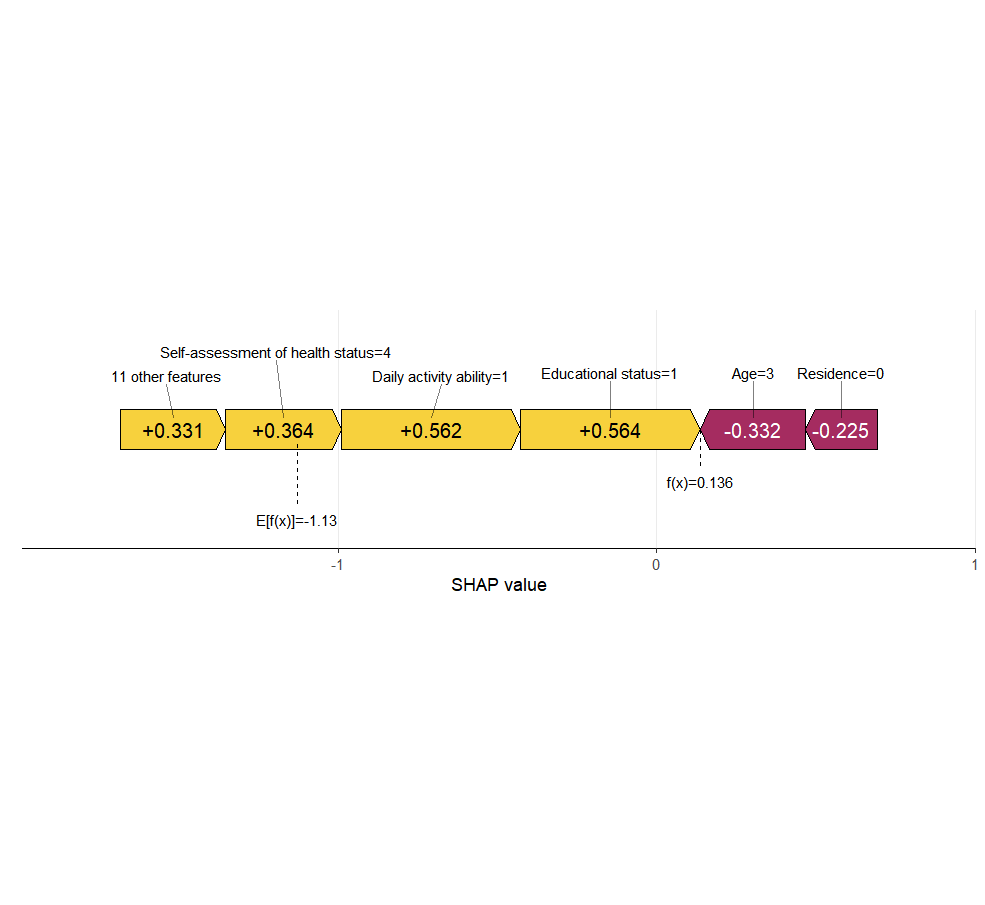

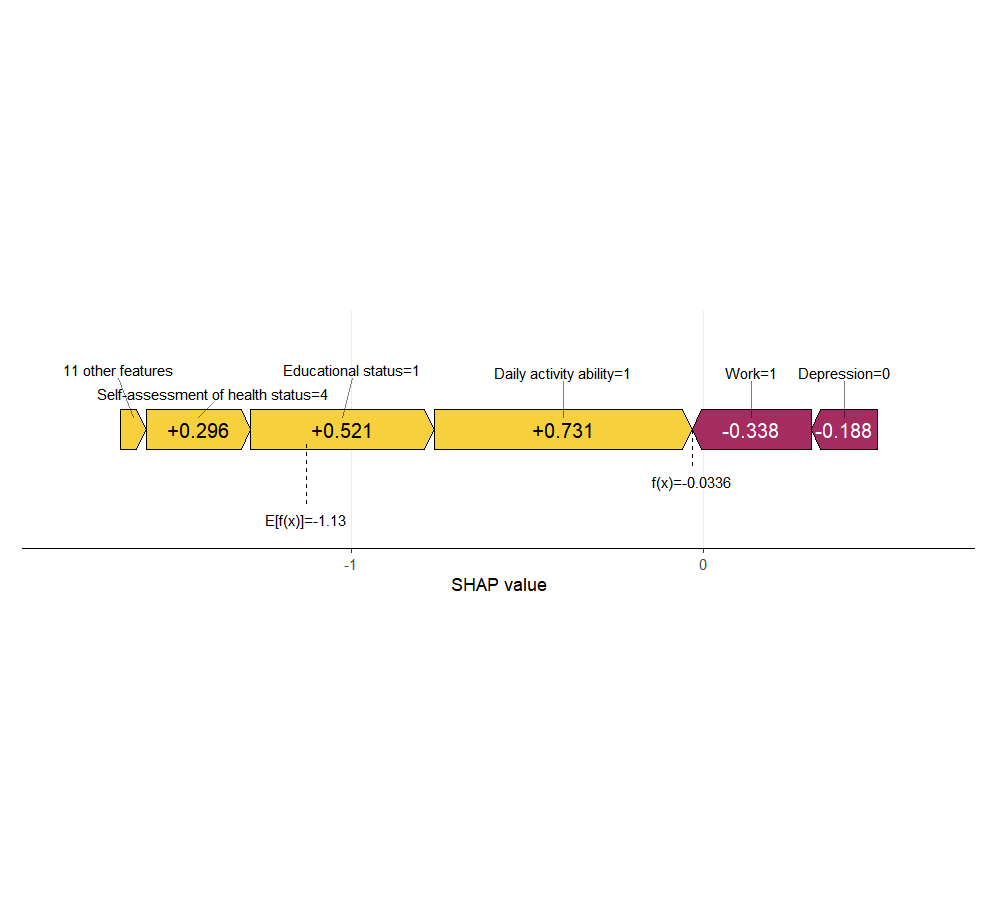


Fig.S5 Subgroup analysis results for the gender group in male based on the XGboost model


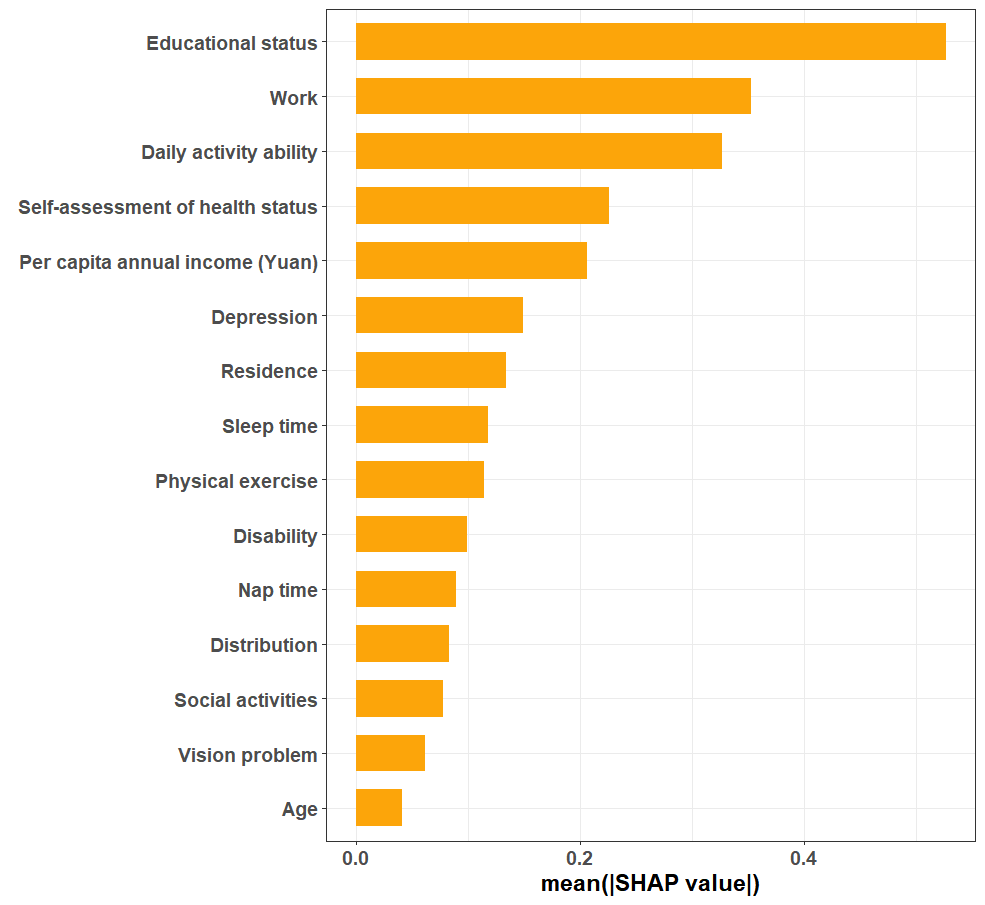

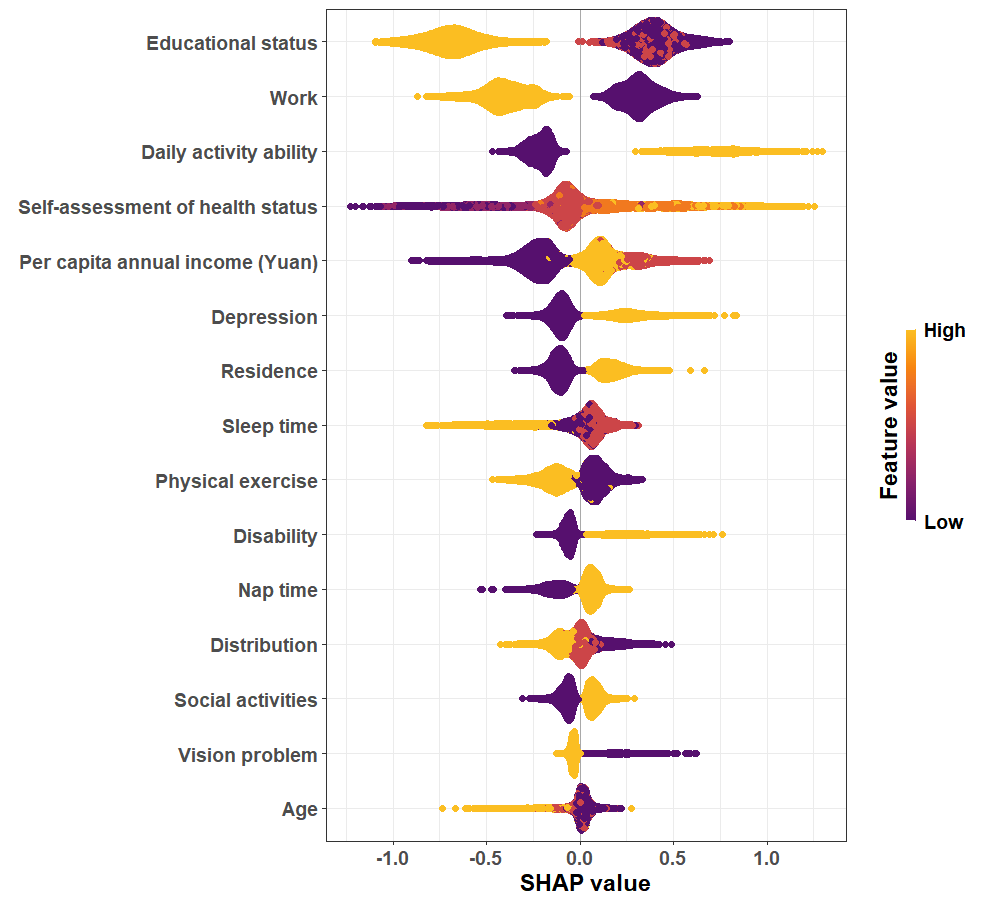

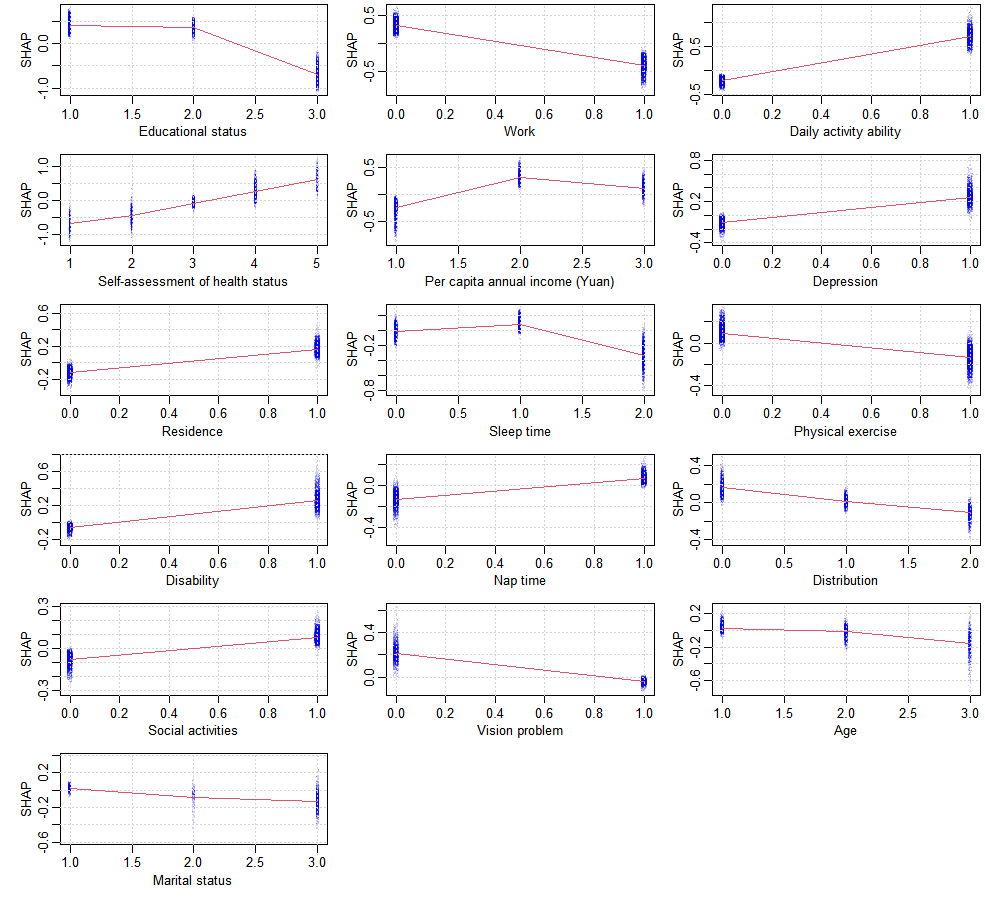

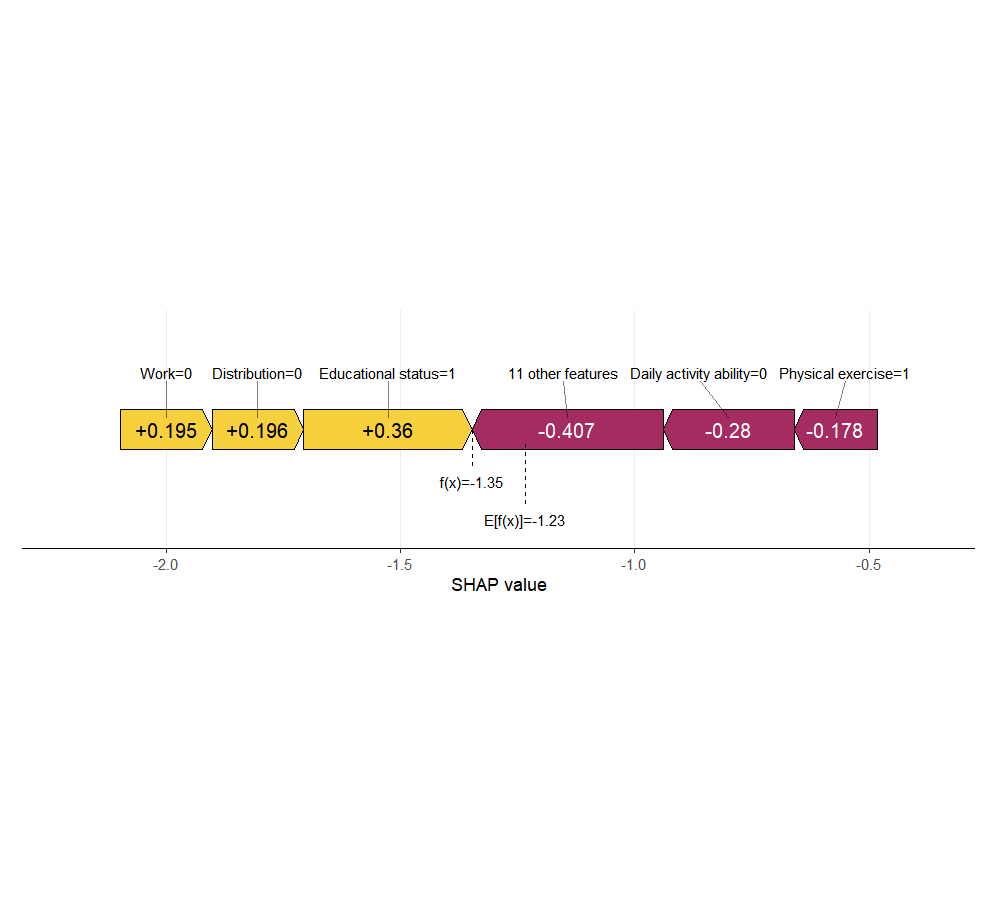

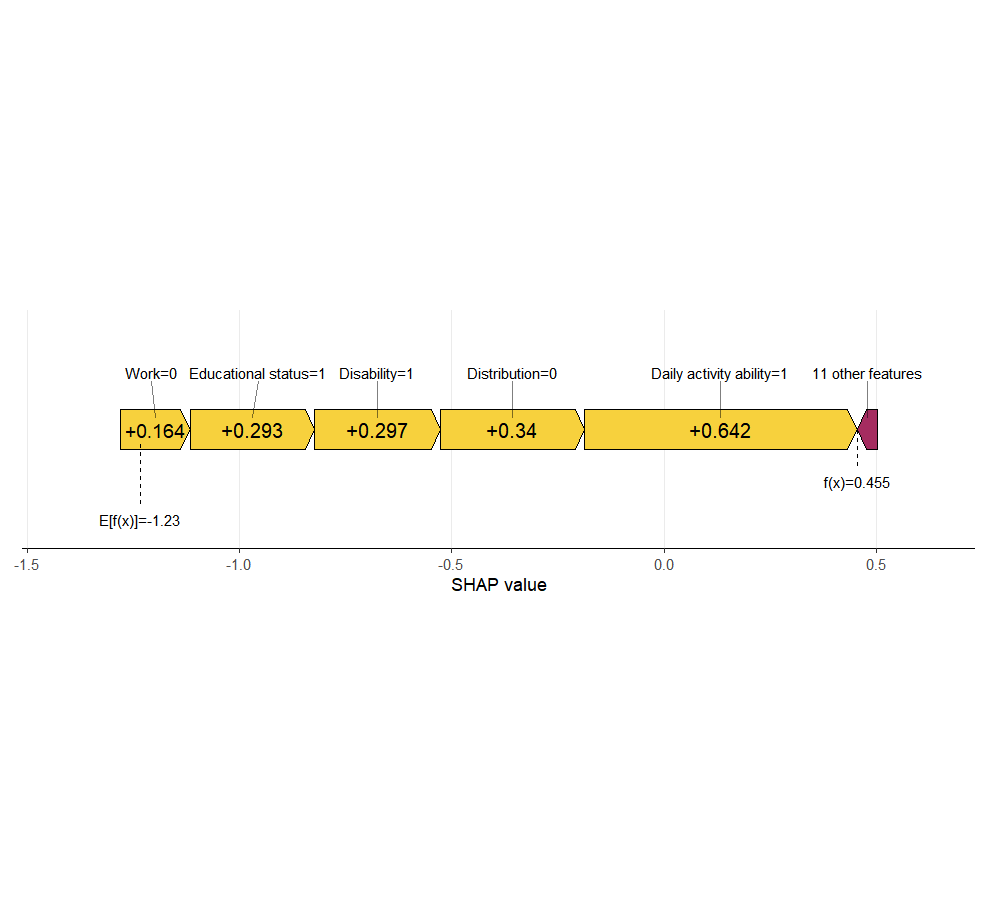


Fig.S6 Subgroup analysis results for the gender group in female based on the XGboost model
